# Internal genes of a highly pathogenic H5N1 influenza virus determine high viral replication in myeloid cells and severe outcome of infection in mice

**DOI:** 10.1371/journal.ppat.1006821

**Published:** 2018-01-04

**Authors:** Hui Li, Konrad C. Bradley, Jason S. Long, Rebecca Frise, Jonathan W. Ashcroft, Lorian C. Hartgroves, Holly Shelton, Spyridon Makris, Cecilia Johansson, Bin Cao, Wendy S. Barclay

**Affiliations:** 1 China-Japan Friendship Hospital, Capital Medical University, Beijing, China; 2 Section of Virology, Department of Medicine, Imperial College London, London, United Kingdom; 3 Section of Respiratory Infections, National Heart and Lung Institute, Imperial College London; 4 Department of Respiratory Medicine, Capital Medical University; Center for Respiratory Diseases, Department of Pulmonary and Critical Care Medicine, China-Japan Friendship Hospital, Beijing, China; University of Georgia, UNITED STATES

## Abstract

The highly pathogenic avian influenza (HPAI) H5N1 influenza virus has been a public health concern for more than a decade because of its frequent zoonoses and the high case fatality rate associated with human infections. Severe disease following H5N1 influenza infection is often associated with dysregulated host innate immune response also known as cytokine storm but the virological and cellular basis of these responses has not been clearly described. We rescued a series of 6:2 reassortant viruses that combined a PR8 HA/NA pairing with the internal gene segments from human adapted H1N1, H3N2, or avian H5N1 viruses and found that mice infected with the virus with H5N1 internal genes suffered severe weight loss associated with increased lung cytokines but not high viral load. This phenotype did not map to the NS gene segment, and NS1 protein of H5N1 virus functioned as a type I IFN antagonist as efficient as NS1 of H1N1 or H3N2 viruses. Instead we discovered that the internal genes of H5N1 virus supported a much higher level of replication of viral RNAs in myeloid cells *in vitro*, but not in epithelial cells and that this was associated with high induction of type I IFN in myeloid cells. We also found that *in vivo* during H5N1 recombinant virus infection cells of haematopoetic origin were infected and produced type I IFN and proinflammatory cytokines. Taken together our data infer that human and avian influenza viruses are differently controlled by host factors in alternative cell types; internal gene segments of avian H5N1 virus uniquely drove high viral replication in myeloid cells, which triggered an excessive cytokine production, resulting in severe immunopathology.

## Author summary

Some avian influenza viruses, including highly pathogenic H5N1 virus, cause severe disease in humans and in experimental animal models associated with excessive cytokine production. We aimed to understand the virological mechanism behind the cytokine storm, and particularly the contribution of internal gene segments that encode the viral polymerase and the non-structural proteins, since these might be retained in a pandemic virus. We found that the internal genes from an H5N1 avian influenza virus allowed virus to replicate to strikingly higher levels in myeloid cells compared to internal genes of human adapted strains. The higher viral RNA levels did not lead to higher viral load but drove excessive cytokine production and more severe outcome in infected mice. The remarkable difference in viral replication in myeloid cells was not observed in lung epithelial cells, suggesting that cell type specific differences in host factors were responsible. Understanding the molecular basis of excessive viral replication in myeloid cells may guide future therapeutic options for viruses that have recently crossed into humans from birds.

## Introduction

The outcome of infection with an influenza virus can vary widely from asymptomatic infection to death. Although infection outcomes can be influenced by host factors such as age, prior immunity, genetic susceptibility and comorbidities [1–4], differences in the virus itself undoubtedly contribute to the variation observed. The most devastating human influenza virus in recent recorded history was the ‘Spanish influenza’ virus that caused the 1918 pandemic. Recombinant influenza viruses reconstructed from the sequences of 1918 influenza are rapidly lethal in animal models [5]. H5N1 ‘bird flu’ is a highly pathogenic avian influenza virus that has occasionally infected humans with catastrophic outcome. Of around 856 people infected by this virus, 52.8% have died. This contrasts starkly with the outcome of infection in 2009 with the new pH1N1 pandemic virus that was associated with just 0.02% case fatality [4,6].

One feature often associated with the severe disease following H5N1 infection, and also noted during animal experimental infections with 1918 virus, is a dysregulated host innate immune response known as the cytokine storm. This response is characterized by excessive levels of inflammatory cytokines and chemokines such as type I interferons (IFNs; IFN-α and β), TNF-α, IL-6, IL-8, CCL2, and CXCL10 [7–11], leading to prolonged fever, lymphopenia, severe pneumonia, and extensive lung damage [12]. Although cytokine storms are not unique to influenza, they are almost always associated with high mortality rates [13–15]. Therefore, it is critical to understand the immunological and virological mechanism behind the activation of these storms, particularly in light of recent evidence that indicates it is possible for H5N1 strains to become airborne transmissible [16–17].

IFNs are important for the antiviral immune response by restricting viral replication. However, excessive type I IFN responses amplify the early pro-inflammatory cytokine response in the lung via type I IFN receptor signaling [18–19], and have been associated with lung inflammation in severe influenza infection [20]. Previous studies revealed that lung epithelial cells, macrophages, conventional dendritic cells (cDCs) and plasmacytoid dendritic cells (pDCs) all express type I IFNs to some extent during influenza infection [21–22]. However, the primary source of type I IFNs in response to influenza infection, especially H5N1, and the virological mechanism behind the cytokine storm remain unknown.

Excessive cytokine production during H5N1 influenza infections occurs in spite of the fact that, like all natural influenza viruses, it encodes a well-known interferon antagonist, the NS1 protein. NS1 suppresses the host’s antiviral response by direct inhibition of activation of RIG-I by viral RNAs [23–24], as well as through dsRNA sequestration, and modification of the expression of induced genes [25–29]. NS1 gene sequences vary between different strains and subtypes of influenza virus and between viruses isolated from different hosts [30]. It is possible the cytokine storm triggered by H5N1 influenza virus in humans is the result of an unadapted avian NS1 protein that does not efficiently antagonize the type I IFN induction pathways in human cells. However, previous reports have shown that NS1 proteins from a variety of different avian influenza virus strains including H5N1 viruses were able to control type I IFN responses in cells of human origin in vitro [31–34].

The severe outcome following HPAI H5N1 influenza virus infection may be, to some extent, dependent on specific genetic features of the H5 HA gene. First, the H5 HA protein harbours a multibasic cleavage site that facilitates multiple cycles of virus replication outside of the respiratory tract. Possession of a multibasic cleavage site in HA determines the highly pathogenic phenotype in poultry hosts [35] and also contributes to the high pathogenicity in the mouse model [36–37]. However, other influenza viruses associated with severe disease including cytokine storm in animal models, such as 1918 H1N1 influenza, do not carry this motif [38]. Other features of the H5 HA that might contribute to H5N1 pathogenicity in mice and humans include a preference to bind α-2,3 sialic acid (SA) receptors thus targeting the virus to the lung [39], a pH of fusion that is higher than for human-adapted strains that might enhance entry into endothelial cells [40], and the ability to trigger acute lung injury through a TLR4 dependent pathway [41]. However, receptor binding specificity and pH stability of H5 HA might be altered if H5 viruses were to gain human transmissibility, so it is important to understand the contribution of internal gene segments to disease severity.

In the following research, we investigated the role of internal genes of an H5N1 influenza virus in the activation of a cytokine storm and compared the virus-host interaction with that of human adapted viruses. To avoid the complication that different viral surface proteins, HA and NA, might affect cell tropism and immune responses *in vitro* and *in vivo*, we rescued a series of viruses that combined a A/Puerto Rico/8/34 (PR8) HA/NA pairing with internal gene segments from either an H1N1, H3N2 or H5N1 virus [36,39,42–45]. Since the PR8 HA and NA genes enable efficient infection of mice, we were able to use mice as a tractable *in vivo* model to study the outcome of infection with the different RG viruses.

Our results reveal that the internal genes of H5N1 facilitate high replication of viral RNAs in haematopoetic cells and drive excessive cytokine production that is the hallmark of infection with these avian influenza viruses.

## Results

### A set of recombinant influenza viruses that differ only in the origin of the internal gene segments replicate with similar kinetics *in vitro* but vary markedly in pathogenicity *in vivo*

**Fig 1:**
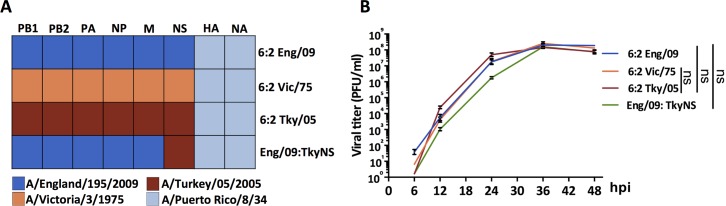
Reassortant influenza viruses rescued by reverse genetic methods (RG). (A) Gene source of the reassortant influenza viruses. (B) Replication kinetics of the reassortant influenza viruses in MDCK cells. Data expressed as mean ± SD (n = 3). Area under the curve (AUC) was calculated for each virus. Difference in the AUC between 6:2 Tky/05 vs. 6:2 Eng/09, 6:2 Tky/05 vs. 6:2 Vic/75, and 6:2 Eng/09 vs. Eng/09:TkyNS was analyzed with one-way ANOVA and Bonferroni’s multiple comparisons test. ns, not significant. hpi, hours post infection.

To address whether the internal gene segments of a highly pathogenic H5N1 avian influenza virus, associated with a fatal human case, contribute to disease severity, we generated a recombinant influenza virus (6:2 Tky/05) with the six internal gene segments from influenza H5N1 A/turkey/Turkey/05/2005 virus (Tky/05) and the HA and NA genes from the laboratory adapted strain PR8. The H5N1 Tky/05 virus is highly pathogenic in mice [46]. In addition, we generated similar recombinant viruses with internal gene segments from a seasonal H3N2 influenza virus A/Victoria/3/1975 (Vic/75) or from a prototypic early pH1N1 pandemic virus A/England/195/2009 (Eng/09), also combined with HA and NA genes from PR8. Finally to assess the importance of the NS gene segment, we generated a chimeric virus with five internal gene segments from the pH1N1 virus (PB1, PB2, PA, NP and M), and the NS segment from the avian H5N1 virus, combined with the PR8 HA and NA genes (Eng/09:TkyNS) (Fig 1A). Thus, all four of these viruses had the same tropism determined by the PR8 HA and NA gene segments. The three 6:2 viruses replicated efficiently in MDCK cells. The virus with Tky/05 internal genes replicated slightly faster than the other two, showing higher titres at 12 and 24 hours. The chimeric virus with Tky/NS gene produced titres around one log less than the other viruses at 12 and 24 hours post infection, although it attained a high titre by 36 hours (Fig 1B).

**Fig 2:**
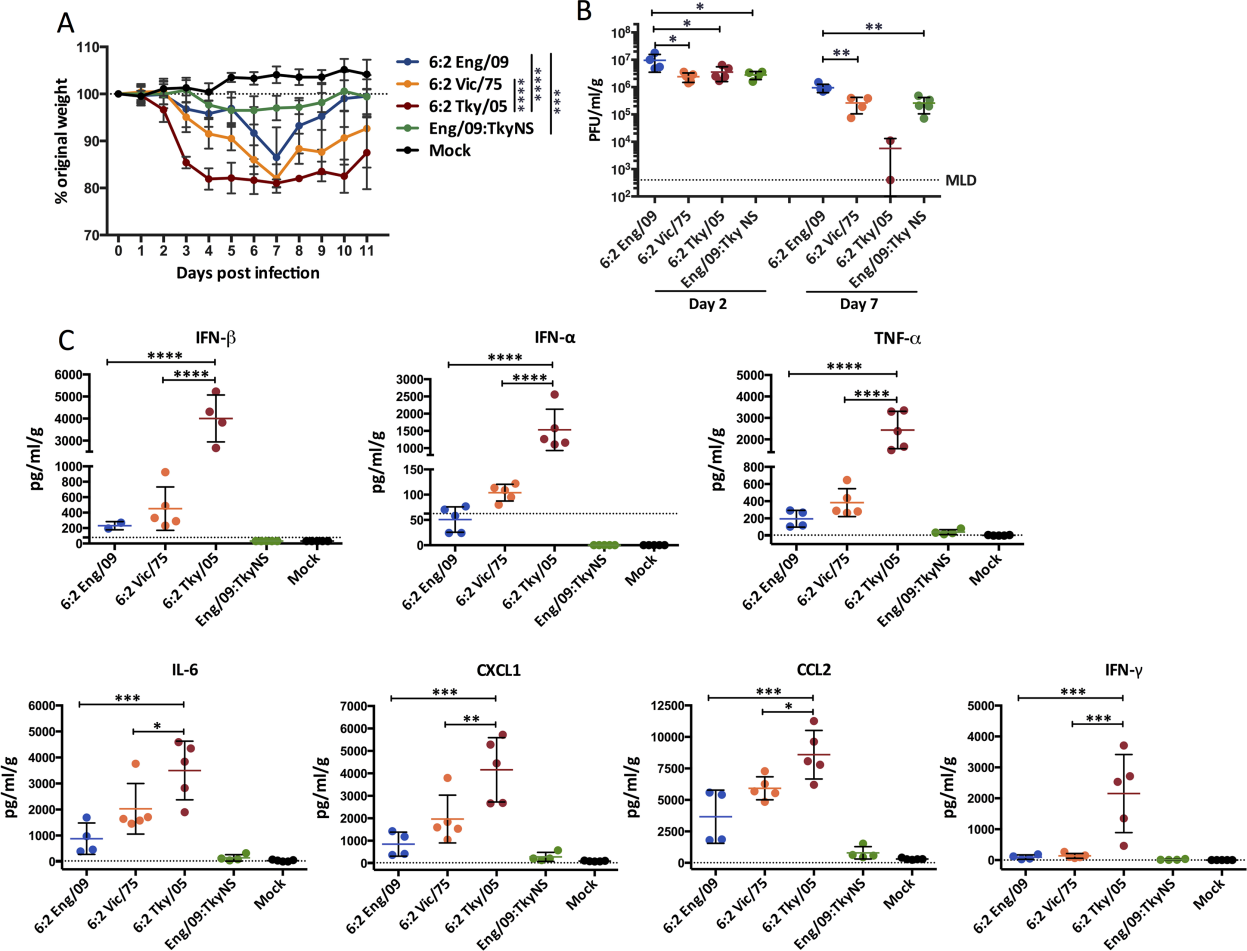
Pathogenicity of the reassortant influenza viruses. (A) Six to eight week old female BALB/c mice (n = 15 per group) were infected i.n. with 10^4^ PFU virus and monitored for weight loss daily. (B) Viral titers in the homogenized lung tissue on day 2 and 7 post infection (n = 5 per group). (C) Cytokine and chemokine analysis of homogenized lung tissue on day 2 post infection Data expressed as mean ± SD (n = 5 per group). The value of 6:2 Tky/05 group was compared with that of 6:2 Eng/09, and 6:2 Vic/75 groups. Most of the cytokines tested in the Eng/09:TkyNS and mock groups are below the minimum level of detection (MLD, the dash line), so were not included in the statistical comparison. *P<0.05, **P<0.01, ***P<0.001, ****P<0.0001.

To assess the pathogenicity of the four RG (reverse genetic rescued) viruses, 6–8 week old BALB/c mice were infected by intranasal inoculation of 10^4^ pfu viruses. All infected mice lost weight over the following 7 days, but weight loss was most dramatic in mice infected with the 6:2 Tky/05 virus (Fig 2A). Mice in this group rapidly lost weight in the first four days after infection, reaching almost 20% weight loss by day 4. Interestingly, this dramatic weight loss did not correlate with a higher lung viral load (Fig 2B) or with increased spread of virus through the lungs (S1 Fig). Indeed, the viral titre in lung homogenates was highest for mice infected with the 6:2 Eng/09 virus on both day 2 and day 7 post infection. Day 2 lung titres were similar between the other three viruses. At the later time point (day 7) two mice surviving in the 6:2 Tky/05 infected group had cleared the virus from the lung but did not regain weight (Fig 2A and 2B).

Instead of viral lung titre, the increased weight loss was associated with an early and high level of cytokines in the lungs of the 6:2 Tky/05 virus infected mice (Fig 2C and S2 Fig). Levels of type I IFNs, TNF-α, IL-6 and CXCL1 as well as CCL2, and IFN-γ were all significantly higher in lungs of 6:2 Tky/05 virus infected mice than in the other three groups at day 2 post infection (Fig 2C).

The dynamics of cytokine detection in the lung tissue (S3 Fig) showed that type I IFNs were produced earlier than most of the other cytokines. IFN-alpha (IFN-α) was detected in the lungs of 6:2 Tky/05 virus-infected mice along with IL-6 at day 2, whereas levels of TNF-α, CXCL1, CCL2 and IFN-γ continued to rise through day 3 post infection.

### 6:2 Tky/05 virus does not induce high type I IFN in epithelial cells and NS1 protein of Tky/05 virus functions as an efficient type I IFN antagonist in these cells

**Fig 3:**
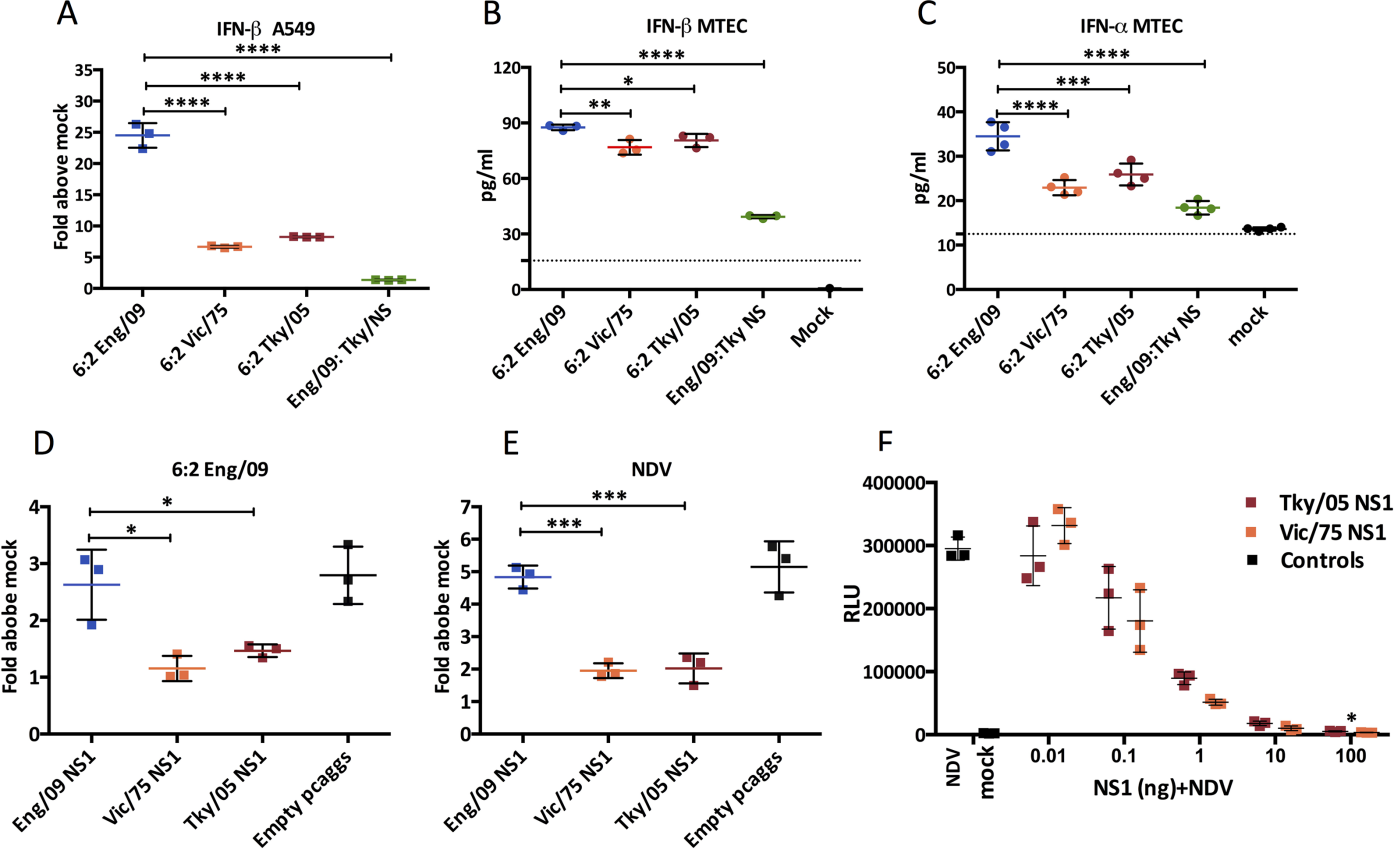
Avian influenza NS1 protein is able to inhibit IFN-β production in A549 cells. (A) Activation of the IFN-β promoter at 24 hpi in A549-Luc cells after infection with 6:2 RG viruses (MOI = 3). (B, C) IFN-β and IFN-α induction at 24 hpi in MTECs (mouse tracheal epithelial cells) after infection with RG viruses (MOI = 3) determined by ELISA. The dashed line indicates the minimum detection limit. (D-F) Suppression of IFN-β promoter in A549-Luc cells by the Eng/09, Vic /75 or Tky/05 NS1 protein. (D, E) 1000 ng plasmid was transfected followed by infection with influenza or NDV. Data are expressed as mean ± SD (n = 3). *P<0.05, **P<0.01, ***P<0.001, ****P<0.0001 indicate significant difference compared between 6:2 Eng/09 and Eng/09:TkyNS virus, and between 6:2 Tky/05, 6:2 Eng/09 and 6:2 Vic/75 NS1; unlabeled indicates no significant difference. hpi, hours post infection.

The most abundantly infected cell type in the virus infected lung is the epithelial cell [47–49]. We previously showed that different influenza viruses varied in the extent to which they induced a type I IFN response in A549 cells, a human lung epithelial cell line [31]. To assess the induction of type I IFNs by the RG viruses in the present study we utilized a reporter A549 cell line we previously generated that harbors a reporter gene with the IFN-β promoter upstream of luciferase. The reporter cells were infected with equal titres of the four RG viruses and luciferase was measured at 24 hours post infection (Fig 3A). In contrast to what was observed *in vivo*, infection with the 6:2 Tky/05 virus did not lead to high activation of the IFN-β promoter. Indeed, it was the 6:2 virus with internal genes from Eng/09 (pH1N1) virus that stimulated the highest luciferase signal in the A549 cells, as we previously described [50]. The 5:1:2 chimeric virus that combined polymerase gene segments from Eng/09 with the NS gene segment from Tky/05 did not induce a high luciferase signal from infected A549 reporter cells, implying that the Tky/05 NS1 protein functioned as an effective type I IFN antagonist (Fig 3A).

We also measured induction of type I IFNs in primary mouse tracheal epithelial cells, MTEC, infected with equal titres of each virus. All four viruses induced IFN-β in a similar pattern to that seen in the human A549 cell line, with the highest levels induced by the 6:2 Eng/09 virus and the lowest by the 5:1:2 Eng/09:TkyNS virus (Fig 3B). IFN-α levels were lower from MTEC cells but showed a similar pattern (Fig 3C).

To compare the ability of the NS1 proteins of each virus to control induction of the IFN-β promoter, we expressed them in the A549 IFN-β reporter cells, and then challenged them with the 6:2 Eng/09 virus, or Newcastle Disease Virus (NDV), to activate the type I IFN induction cascade. We found that the NS1 protein from Tky/05 virus suppressed IFN-β induction efficiently, as did Vic/75 NS1 and both were more effective than the NS1 protein of the Eng/09 virus (Fig 3D and 3E). Indeed a titration of plasmids encoding Vic/75 and Tky/05 NS1 proteins demonstrated no difference in their efficiency to suppress the IFN-β signal (Fig 3F).

### 6:2 Tky/05 virus induces high secretion of type I IFN in innate immune cells *in vitro*

Although epithelial cells are the primary target of infection and the major producers of progeny virus, dendritic cells and macrophages have also been reported to be infected by influenza virus, and are believed to be the main producers of cytokines and type I IFNs *in vivo* [10,20,51–55]. So far there is not a thorough understanding of which is the major cell type that contributes to differences in the extent of type I IFN production induced by infection with different viruses, nor the mechanism that defines such differences.

**Fig 4:**
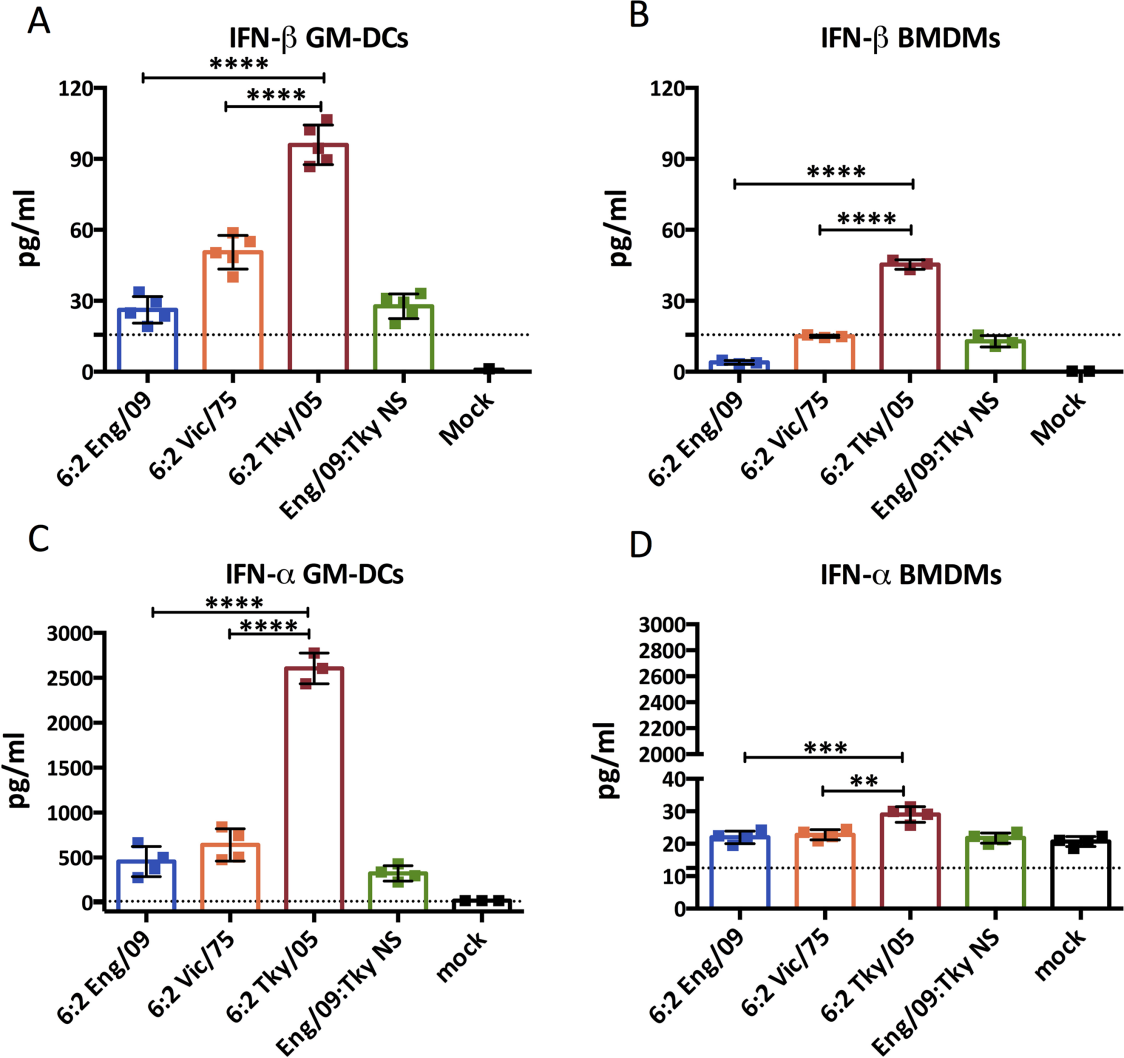
IFN-β and IFN-α induction in myeloid cells after infection with reassortant influenza viruses. IFN-β and IFN-α induction at 24 hpi in GM-DCs (A, C) and BMDMs (B, D) after infection with MOI = 10 of RG viruses determined by ELISA. Data are expressed as mean ± SD (n = 3). The value of 6:2 Tky/05 group was compared with that of 6:2 Eng/09 or 6:2 Vic/75 groups. *P<0.05, **P<0.01, ***P<0.001, **** P<0.0001; unlabeled indicates no significant difference. The dashed line indicates the minimum detection limit. hpi, hours post infection.

We aimed to find a cell population that could be studied *in vitro* that reflected the pattern of type I IFNs produced by our panel of viruses *in vivo*. To this end we propagated bone marrow derived cells in different media to produce populations with phenotypes similar to macrophages (BMDMs, propagated with L929 conditioned media), dendritic cells (BMDCs propagated in GM-CSF termed GM-DCs) or plasmacytoid dendritic cells (BMDCs propagated with Flt3 ligand termed FL-DCs). Each cell population was infected at equal multiplicity with the RG influenza viruses. Strikingly, IFN-α and IFN-β production from GM-DCs and IFN-β production from BMDMs propagated using L929 conditioned media, reflected the same pattern as seen *in vivo*, in that type I IFN levels were significantly higher from cells infected with the 6:2 Tky/05 virus than for any of the other viruses (Fig 4). In addition to the high type I IFN responses observed, the GM-DCs infected *in vitro* with 6:2 Tky/05 virus, but not the other viruses, also displayed a high induction of TNF-α and IL-6 mRNAs (S4 Fig). In contrast, FL-DCs produced very low levels of IFN-β upon infection. Levels of IFN-α after infection of FL-DCs were higher but were not different between 6:2 Vic/75 and 6:2 Tky/05 viruses (S5 Fig).

### Increased virus RNA replication in GM-DCs accounts for the high level of IFN-α production by 6:2 Tky/05 virus

**Fig 5:**
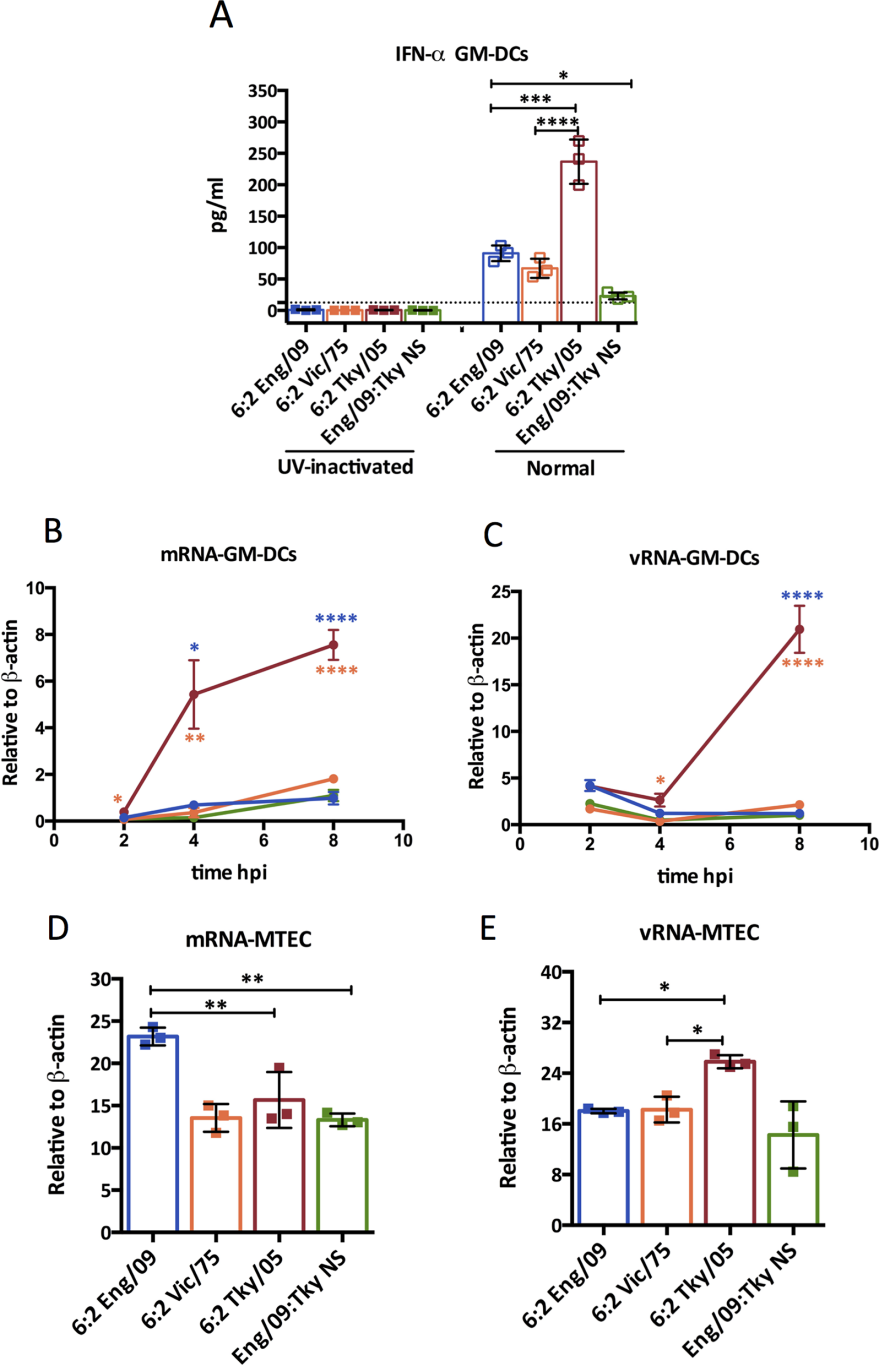
IFN-α production from GM-DCs requires viral replication. (A) IFN-α induction at 24 hpi in GM-DCs infected with RG viruses at MOI = 10 or exposed to the same viruses after UV-inactivation. Bone marrow derived GM-DCs (B, C) and MTEC (D, E) were infected with the indicated virus at MOI = 10 and 3, respectively. Total RNA from the GM-DCs at 2 hpi, 4 hpi or 8 hpi (B, C) and from MTEC at 8hpi (D, E) was extracted. RT-PCR was performed using specific primers targeting segment 4 (HA) for each species of virally derived RNA. Bars represent mean ± SD (n = 3). The value of 6:2 Tky/05 group was compared with that of 6:2 Eng/09 or 6:2 Vic/75 groups, and the difference between the 6:2 Eng/09 and Eng/09:TkyNS was also assessed. *P<0.05, **P<0.01, ***P<0.001, ****P<0.0001; unlabeled indicates no significant difference. The dashed line indicates the minimum detection limit. hpi-hours post infection.

We next carried out experiments to understand the basis of the high type I IFN induction observed *in vitro* in infected GM-DCs. We found that IFN-α production induced by infection with any of the recombinant influenza viruses depended on virus replication since there was no signal following UV inactivation of input virus (Fig 5A).

To test the hypothesis that high levels of RNA generated during replication by the 6:2 Tky/05 virus drove the high type I IFN response in myeloid cells, we measured the accumulation of viral RNAs by qRT-PCR in different cell types. In GM-DCs, by 8 hours post infection levels of mRNA and vRNA of the Tky/05 virus were 7.7 and 17.0 fold higher than that of the human adapted viruses, respectively (Fig 5B and 5C). In contrast, in MTEC levels of viral RNAs were not higher for the Tky/05 virus (Fig 5D and 5E). In other epithelial cells tested, human A549 cells or mouse LA4 (lung epithelial) cells, there was also little or no difference between levels of viral RNAs produced (S6 Fig). At 8 hours post infection of LA-4 cells the Tky/05 virus produced just 2 fold more vRNA than the other viruses (S6 Fig).

**Fig 6:**
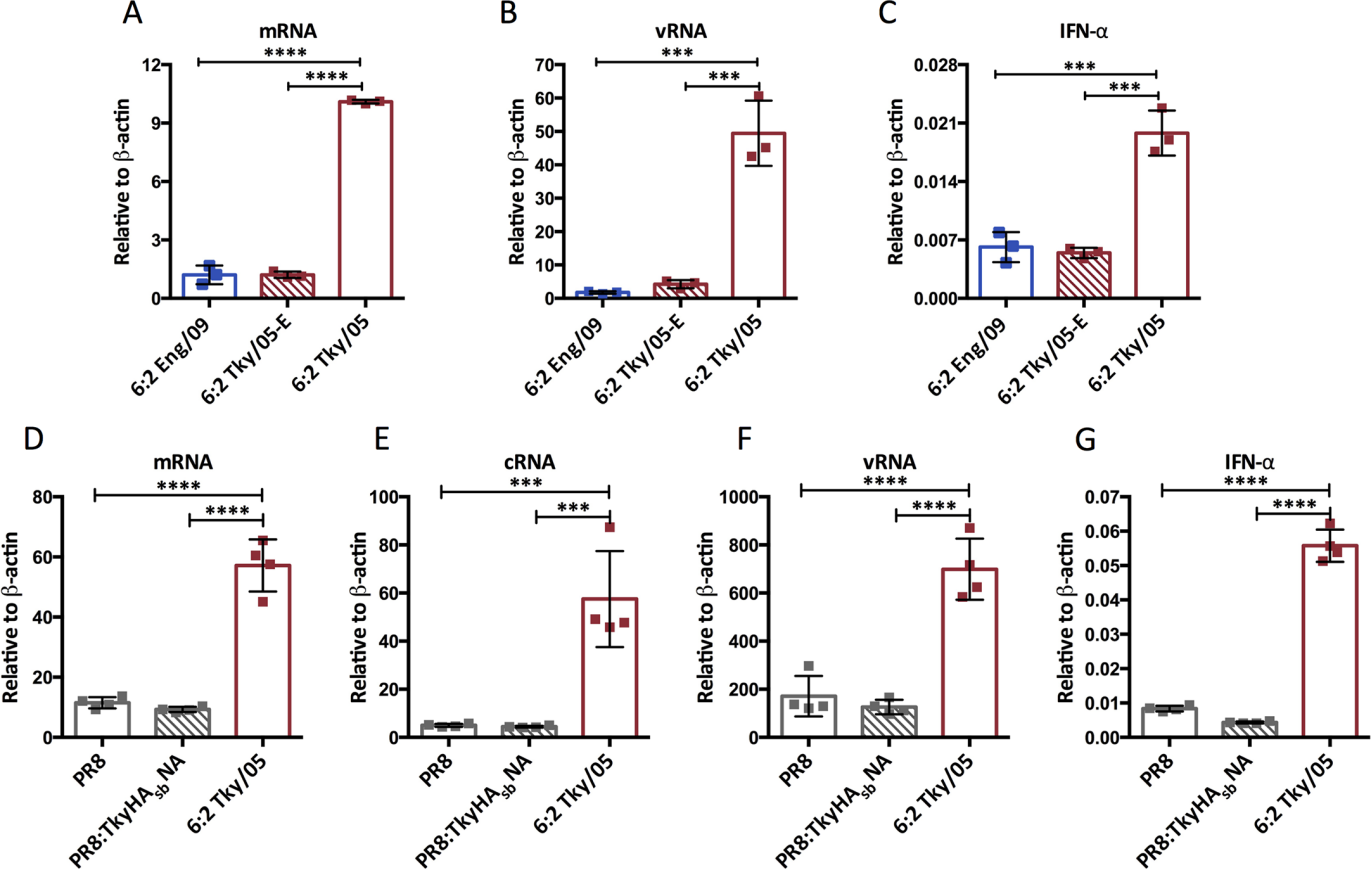
Higher replication and IFNα induction in GM-DCs of the 6:2 Tky/05 virus depends on mammalian adaptation of RdRp and was not seen for virus with internal genes from PR8. Bone marrow derived GM-DCs were infected with the indicated virus at MOI = 10. Total RNA was harvested at 8 hpi. RT-PCR was performed using primers targeting segment 4 (HA) (A-C) and segment 7 (M) (D-G) respectively. Bars represent mean ± SD (n = 3). Differences between groups were analyzed with one-way ANOVA and Bonferroni’s multiple comparisons test. *P<0.05, **P<0.01, ***P<0.001, ****P<0.0001; unlabeled indicates no significant difference.

We also generated a mutant virus that would be compromised in replication in mammalian cells by engineering the mutation K627E in the Tky/05 PB2 gene segment. Indeed accumulation of v and mRNAs in GM-DCs infected with this virus were greatly decreased compared to the ‘wild type’ 6:2 Tky/05 virus with the mammalian-adapting 627K motif (Fig 6A and 6B). The mutant virus with PB2 627E no longer induced IFN-α mRNA in infected GM-DCs (Fig 6C). In order to ensure the PR8 HA and NA of the Ty/05 virus was not misrepresenting the ability of an H5N1 virus to infect and replicate in GM-DCs, we generated PR8:TkyHA_sb_NA, that contained the six internal genes from PR8 and the H5 HA and N1 NA from A/turkey/Turkey/05/2005 virus. To make it biologically safe, the multi-basic cleavage site of H5 HA was removed. We compared the infection of GM-DCs by this virus with infection by whole PR8 virus and found them to be similar (S7 Fig). The m, c, and vRNA accumulation were also similar between PR8 and PR8:TkyHA_sb_NA infected GM-DCs, and significantly lower than in GM-DCs infected with 6:2 Tky/05 virus (Fig 6D–6F). Accordingly, neither the PR8:TkyHA_sb_NA virus nor the whole PR8 virus induced high IFN-α expression (Fig 6G).

To probe the pathway that led to high type I IFN induction in GM-DCs, we infected GM-DCs derived from *Mavs*^-/-^ mice and assessed the IFN-α response. In the absence of MAVS, none of the viruses induced a type I IFN response despite there being high levels of viral RNA following infection with the 6:2 Tky/05 virus (S8 Fig).

### Cells of haematopoetic origin are a source of IFN-α in 6:2 Tky/05 virus infected mice

To confirm that cells of haematopoetic origin contribute to the excessive IFN-α *in vivo* in 6:2 Tky/05 virus infected mice, we FACS purified CD45-positive cells from lungs harvested 2 days post infection and performed qRT-PCR for viral RNAs and for IFN-α and IL-6 mRNAs. We found evidence of vRNA and mRNA in this cell population as well as a type I IFN and IL-6 cytokine response (S9 Fig).

**Fig 7:**
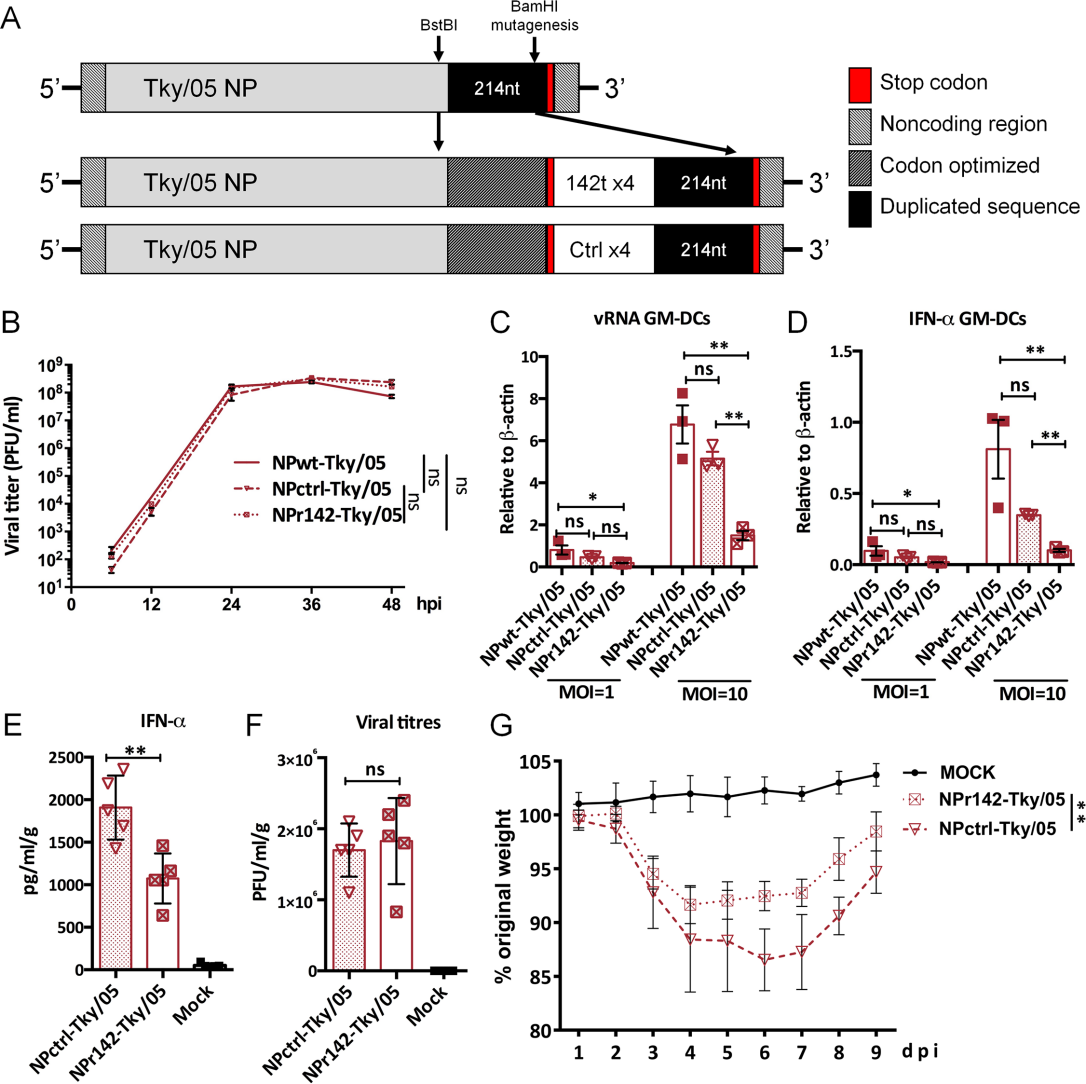
Inhibition of viral replication of 6:2 Tky/05 in haematopoetic cells decreased both IFN-α production in lung and disease severity. (A) Schematic of the NP segment of the engineered viruses. (B) Replication kinetics of the NPwt-Tky/05, NPctrl-Tky/05, and NPr142-Tky/05 viruses in MDCK cells. Viral replication (C) and IFN-α induction (D) in GM-DCs 24 hpi, quantified with SYBR Green. Values were calculated by the 2^ΔCt^ method. Six to eight week old female BALB/c mice (n = 10 per group) were infected i.n. with 10^5^ PFU virus and weight loss was monitored daily (G). IFN-α production (E) and viral titers (F) in lung tissue on day 2 post infection. * P<0.05, ** P<0.01, ***P<0.001, ****<0.0001; ns, not significant. In Fig E-G, only significant differences between NPctrl-Tky/05 and NPr142-Tky/05 virus infected mice were labeled.

To further demonstrate that cells of hematopoetic origin were important in the cytokine response and severe outcome of infection with the 6:2 Tky/05 virus, we engineered a recombinant virus (NPr142-Tky/05) that harboured four copies of a microRNA target sequence in the NP gene for a microRNA specifically expressed in cells of haematopoetic origin, MiR142, as previously described by Langlois et al [56]. This would result in cell type specific reduction in virus replication since levels of NP protein required to support replication would be specifically reduced in myeloid cells. A control virus contained an inserted sequence at the same location that was not a MiR target (NPctrl-Tky/05) (Fig 7A). NPr142-Tky/05 and NPctrl-Tky/05 viruses showed similar replication in MDCK cells (Fig 7B). However, in GM-DCs, vRNA accumulation following infection with the virus containing MiR142 target sites was significantly reduced compared to control virus (Fig 7C) and this led to a decreased level of IFN-α mRNA in these cells (Fig 7D).

Mice were infected with 10^5^ pfu of each recombinant virus, and monitored for weight loss, lung titre and IFN-α in lung homogenates at day 2 (Fig 7E–7G). The insertion of the MiR142 target sequence, that reduced the extent of replication in cells of hematopoietic origin, resulted in reduced weight loss that correlated with a lower IFN-α level in the lung (Fig 7E and 7G). Virus titre in the lung was not affected (Fig 7F), suggesting that the majority of infectious virus is produced from epithelial cells, but that replication in cells of haematopoetic origin is the source of the excess type I IFN produced by the 6:2 Tky/05 virus.

Finally, we attempted to attribute the high cytokine inducing phenotype to a particular viral polymerase or NP gene by creating a set of recombinant viruses based on the 6:2 Tky/05 virus in which each RNA segment was exchanged for that from the Eng/09 virus (S10A Fig). Although several of these viruses replicated to high titres in mouse lung, none induced rapid weight loss as seen for the 6:2 Tky/05 virus (S10B and S10C Fig). Furthermore, exchanging any one polymerase or NP segment for that of Eng/09 abrogated the high cytokine induction in the lungs of infected mice suggesting that a single gene of the Tky/05 virus is not responsible for the high cytokine phenotype but rather the particular replication activity of the Tky/05 polymerase and NP complex. (S10D Fig).

## Discussion

The severity of the next influenza virus pandemic will be determined by the nature of the virus that emerges from an animal source and acquires an airborne transmissible phenotype. The HPAI H5N1 virus has been a public health concern for more than a decade because of frequent zoonoses and the high case fatality rate associated with human infections [57–59]. At least part of the high virulence of this virus can be explained by features of the H5 HA protein such as a propensity to bind to α-2,3-SA receptors in the lungs, an ability to enter endothelial cells, and a multi-basic cleavage site that might enable systemic spread and infection of cell types not usually infected by seasonal influenza viruses [35,42,60–63]. Indeed recent work by Tundup *et al*. used a similar approach to that employed here to control virus tropism *in vivo* by engineering MiR target sites into RG H5N1 virus, and highlighted the importance of endothelial cell infection in H5N1 pathogenesis in mice [37]. However, some features of the H5 HA that support the extended tropism of H5N1 avian influenza would likely be lost if the virus gained airborne transmissibility, and so it is important to understand how other genes of the virus also affect pathogenesis if we are to predict the likely severity of an H5N1 pandemic.

Although the ferret model is considered the gold standard for influenza infection and transmission, the HA/NA pairing employed in these studies to normalise viral entry would not facilitate significant viral replication in the ferret respiratory tract due to receptor specific incompatibilities [64]. Therefore, we focused on the mouse model of influenza. In contrast to the ferret model, the mouse model of influenza benefits from a plethora of established protocols and immunological reagents, as well as a fully annotated genome. In particular, the lack of annotation of the ferret genome makes it uncertain as to whether the NPr142-Tky/05 virus would be restricted in hematopoetic cells. A recent article, as well as our own nucleotide BLAST analysis, have revealed a high degree of similarity between mouse miRNA-142 and an unannotated miRNA in the ferret genome, but further research is necessary to understand whether this putative miRNA-142 displays similar cell-type specific effects in ferrets as it does in mice [37].

Here we assessed the contribution of internal gene segments that encode the viral RNA dependent RNA polymerase (RdRp), nucleoprotein, matrix and nonstructural proteins to the outcome of H5N1 influenza virus infection. Using a reverse genetics strategy, we engineered viruses that had an identical ability to bind and enter cells because they encoded the same HA/NA pairing, but differed in their interaction with factors inside the infected cells depending on the human or avian virus origin of the segments encoding the internal genes. This revealed that the internal genes of the H5N1 virus contribute to the severe outcome *in vivo*. Upon infection of mice with the virus with H5N1 internal genes, dramatic body weight loss was accompanied by high levels of type I IFNs and other inflammatory cytokines in the lungs, although titres of virus were not higher than in mice infected with human adapted viruses. Two previous studies also found that the polymerase genes of H5N1 influenza virus determined the high cytokine response in human macrophages and in mice but did not solve the underlying mechanism [65–66].

Recent microarray analysis has confirmed prior genetics and biochemistry studies, which implicate type I IFNs as the main driver of many other cytokines during influenza infection [67]. However, the cell type that is the primary source of type I IFN, especially during H5N1 infection *in vivo* was not clear. Previous work has shown that CD11c+ cells, which constitute macrophages, monocytes and dendritic cells, produce the bulk of IFN-β *in vivo* following infection with a mouse adapted influenza virus [51]. Our data suggest that H5N1 triggers an unusual and excessive cytokine response compared to human adapted viruses in cells of haematopoetic origin (Figs 4–6). *In vitro*, using GM-DCs, which are thought to represent a mixture of monocyte derived and conventional DCs [68], we found that the 6:2 H5N1 virus induced significantly more type I IFNs than the equivalent human adapted viruses. In contrast, in epithelial cells, the 6:2 H5N1 virus was not a potent type I IFN inducer. Conflicting reports in the literature about the propensity of H5N1 influenza viruses to trigger high type I IFNs might be explained by our cell type specific findings: Cheung *et al*. first reported high cytokines induced by H5N1 viruses using human monocyte derived macrophage populations, whereas Zeng *et al*. found that H5N1 viruses effectively controlled type I IFNs in human bronchial epithelial cells [10,69]. Although GM-derived dendritic cells are not perfect analogues for dendritic cells found in the lung, it is striking that the pattern of type I IFNs production in these cells closely resembled that produced *in vivo* during infection by the three strains of RG 6:2 influenza viruses. Moreover the decrease in lung type I IFN levels during infection with the Mir142 targeted RG virus confirms that a proportion of the cytokines that contribute to the proinflammatory response during H5N1 virus infection were secreted *in vivo* by infected haematopoetic cells.

One explanation for the high levels of type I IFNs during H5N1 virus infection would be that virally encoded IFN antagonists from the avian derived Tky/05 virus did not control the type I IFN response in mammalian cells. Three pieces of evidence from our study ruled out that the H5N1 NS1 protein was deficient in this regard: first, there were low levels of cytokines in lungs of mice infected with the 5:1:2 Eng/09:TkyNS virus, second, the 6:2 Tky/05 virus effectively controlled type I IFN induction in epithelial cells and third, exogenously expressed Tky/05 NS1 protein could efficiently suppress an IFN stimulus, at least in epithelial cells. Since several other viral gene products are implicated in controlling the innate immune response, including two of the polymerase proteins, PB2 and PA, as well as the accessory proteins PB1-F2, and PA-X, it may be that one or more of these functions are defunct in the Tky/05 virus, although sequence analysis based on current knowledge does not support this [70]. Perhaps some IFN antagonists of the avian influenza virus do not function in mammalian myeloid cells. We were not able to test directly whether exogenously expressed Tky/05 NS1 protein or any other virally encoded IFN antagonists were able to control type I IFN induction in the GM-DC cells we employed here. However, using a panel of RG viruses in which individual polymerase gene segments were swapped between Tky/05 and Eng09, we did not find any single viral gene to which we could attribute the high cytokine inducing phenotype (S10 Fig). The alternative and more plausible explanation for our data is that the higher levels of a viral RNA species produced during H5N1 infection in myeloid cells, which are more sensitive to viral pathogen associated molecular patterns (PAMPs) than epithelial cells, outweighed the potency of the H5N1 virus-encoded IFN antagonists.

We suggest that the PAMP responsible is a replication product of the viral polymerase. We found that viruses with H5N1 internal genes had an unusual propensity to drive high levels of RNA replication in the GM-derived DC cells infected *in vitro*. The conventional pathway by which influenza virus triggers a type I IFN response is by RIG-I detection of replicated viral RNAs and subsequent signaling through MAVS [71–73]. Infection of GM-DCs derived from *Mavs*^-/-^ mice did not result in any type I IFN production despite similar levels of virus replication (S8 Fig) suggesting the pathways triggered by H5N1 virus are the conventional ones. Type I IFN induction in MAVS positive cells was dependent on replication and correlated with the amount of viral RNAs generated. We hypothesize that one or more host cell factors that are differentially expressed in myeloid cells vs. epithelial cells affect the behavior of the RNA dependent RNA polymerase (RdRp) of the avian and the human adapted virus differently. Thus the severe disease resulting from infection with H5N1 virus stems from excessive RNA polymerase activity in mammalian myeloid cells. High viral replication has been previously linked with the clinical outcome of H5N1 infection in mice [36]. The inappropriate early type I IFN response is not dampened by viral IFN antagonists and drives a downstream production of inflammatory cytokines in the lung that leads to severe weight loss. Whether the lack of control of virus replication in myeloid cells is a common feature of other avian influenza viruses associated with severe human infections, whether there is a specific virus signature responsible and the identity of host factors that determine these differences, remain to be established.

## Materials and methods

### Cells, plasmids and viruses

LA4 cells were acquired from Prof. Robert Snelgrove (Leukocyte Biology Section, National Heart and Lung Institute, Imperial College London) and were originally obtained from American Type Culture Collection (ATCC). LA4 cells were maintained in Ham’s F12 medium conditioned with 2mM L-glutamine and 15% FBS. Human embryonic kidney (293T) (ATCC), human lung adenocarcinoma epithelial cells (A549) (ATCC) and Madin-Darby canine kidney (MDCK) cells (ATCC) cells were maintained in Dulbecco’s modified Eagle’s medium (DMEM; Gibco, Invitrogen) supplemented with 10% FBS and 1% penicillin/streptomycin (Sigma-Aldrich). A549-Luc cells were constructed and maintained as previously described [74]. L929 cells were a gift from Caetano Reis e Sousa, The Francis Crick Institute, UK, and were cultured with 10% FBS, 0.05mM β-mercaptoethonal and 1% penicillin/streptomycin in conditioned RPMI 1640 medium.

Plasmids used in this study to rescue viruses have been previously described [75–76]. NS1 expression plasmids utilized in Fig 3D–3F were described previously[50].

All the viruses used in this research were rescued by reverse genetics. Briefly, 12 plasmids, comprising of 8 polI plasmids encoding the indicated virus segments and 4 helper expression plasmids encoding A/Victoria/3/75 polymerase components and NP expressed by the pCAGGS vector, were transfected into the 293-T cells that were then co-cultured with MDCK cells. Virus stocks were grown on MDCK cells using serum free DMEM supplemented with 1ug/mL of TPCK trypsin. Viruses were stored in -80°C and titrated on MDCK cells by plaque assay.

### NP-r142, NP-ctrl plasmid design

The modified Tky-NP segment was generated by PCR and ligation. We added a BamHI site 62bp upstream of the 3’ terminus of the complimentary sequence by site directed mutagenesis. Utilizing a naturally occurring BstBI restriction site 257bp upstream of the 3’terminus, we were able to insert a synthesized 478bp-length BamHI and BstBI flanked sequence (GeneArt). The sequence contained 231bp of codon optimized open reading frame upstream of the stop codon, four tandem copies of miRNA142 target sequence or scrambled control sequence, and 146bp duplicated segment 5 packaging sequence. Thus we created a plasmid, which conserved both the amino acid composition of the NP protein and the packaging sequence of the segment [56,77]. The miRNA142 target was the same as previous described [56]. The scrambled control sequence was provided by Lucy Thorne (University College London): AGACAATACGTCACATATAACGA. The modified NP plasmids and RG virus were sequence verified.

### Growth kinetics of viruses in cells

MDCK cells were infected with the indicated virus at MOI = 0.001 and overlaid with serum free DMEM containing 1μg/ml TPCK trypsin. Supernatants were collected at a given time point after infection and stored at -80°C. Virus was titrated by plaque assay in MDCK cells.

### Inactivation of viruses

Inactivation of virus was performed by UV radiation. Indicated viruses were exposed on ice to short wave UV radiation at 254nm for 10 min at a distance of 15cm. Virus integrity and loss of infectivity was confirmed by Haemagglutination test with chicken erythrocytes (Envigo RMS (UK) Ltd) and plaque assay, respectively.

### Plaque assay and Haemagglutination assay

Plaque assays were performed as previously described [76]. Briefly, 100% confluent MDCK cell monolayers were inoculated with 100 μl of serially diluted virus and overlaid with 2% agarose (Oxoid) in supplemented MEM with 2.6 μg/ml trypsin (Worthington) and incubated at 37°C for 3 days.

For Haemagglutination assay, 0.5% chicken erythrocytes (Envigo RMS (UK) Ltd) were mixed with serially diluted virus in a 96-well plate. After half an hour’s incubation on ice, the results were read by eye.

### A549 transfection with NS1 and viral infection

A549-IFNβ cells were transfected with NS1-pCAGGS plasmid. Briefly, fully confluent cells were incubated with 1000ng/well of either Eng/09, Vic/75, or Tky/05 NS1-pCAGGS plasmid in Opti-MEM and lipofectamine (Invitrogen) for 2–4 hours. Opti-MEM was removed and replaced with 10% DMEM overnight. Viral challenge of transfected cells was performed at either an MOI 3 (influenza infections), or with a final dilution of 1:100 (NDV). Eighteen hours post infection, cells were lysed and harvested with 200μl passive lysis buffer (Promega), then assayed for luciferaseusing FLUOstar Omega Plate Reader (BMG Labtech).

### Mice and infections

Six to eight week old female BALB/c or C57B6 mice (Charles River UK Ltd or Envigo RMS UK Ltd) were maintained in pathogen-free conditions until used for viral infection or cell isolation. *Mavs*^-/-^ mice were a kind gift from Professor S. Akira [18,78].

Mice were infected intranasally with the indicated PFU of virus or serum free DMEM (mock) in a 25ul volume under isofluorane. Animals were monitored and weighed daily. Lungs were harvested on Day 1, 2, 3, 4, or 7, or when weight loss dropped below 80% of the original weight on Day 0. Lungs were suspended in 1ml of PBS and homogenized using 1.3mm beads, homogenates were frozen in -80°C before testing the virus titer and cytokine expression.

### Generation of mouse bone marrow derived GM-DC, FL-DC, and macrophages

To isolate bone marrow cells, femur and tibia of three-to-six week old female BALB/c mice were excised and cleaned of flesh. Bone marrow cells were flushed out, filtered through a nylon cell strainer (Falcon, 2350) and washed with PBS. Cells were resuspended and differentiated in RPMI 1640 medium (10% FBS, 0.05mM β-mercaptoethanol and 1% penicillin/streptomycin) supplemented with GM-CSF (R&D, cat 415-ML-010: final concentration 40 ng/ml), or Flt3-L (R&D, cat 427-FL-025: final concentration 250 ng/ml), or 20% L-929 cell supernatant. On day 3 or 4 of culture, non-adherent cells, which are mostly granulocytes, were removed and fresh medium containing the same concentration of GM-CSF or Flt3-L or L929 cell supernatant was added. On day 7, cells were harvested for further experimental use [79–81].

### Primary mouse tracheal epithelial cell culture (MTEC)

Mouse tracheal cells were isolated from three-to-six week old female BALB/c mice by pronase digestion. The cells were seeded in a petri dish and incubated for 3–4 hours to adhere fibroblasts. Then non-adherent cells were collected and reseeded in a 10cm petri dish and cultured with DMEM/F12 medium supplemented with 10% FBS, 15 mM HEPES (Gibco), 0.03% NaHCO3 (Gibco), 0.01uM Retinoic acid (Sigma, cat R-2625), Amphotericin B (Gibco, cat A2942: final concentration 250ng/ml), EGF (BD, cat 354001: final concentration: 25ng/ml), D-valine (Sigma, V1255: final concentration: 0.1mg/ml), bovine pituitary extract (Gibco, cat 13028–014: final concentration: 30 ug/ml), Cholera Toxin (Sigma, cat C8052: final concentration: 0.1ug/ml), Insulin-Transferrin-Selium (Gibco, cat 41400045: 1:100). Medium was changed every two days. After 14 days incubation and differentiation, the epithelial cells were harvested for experimental use.

### Measurement of viral RNA from infected cells

Bone marrow derived GM-DCs, FL-DCs and macrophages, LA4, A549, or MTEC cells were seeded on 96-well plates (about 1.25×10^5^ cells/well) and infected with virus diluted in serum free DMEM for 1hr at 37°C (MOI as indicated in the relevant figure legends) and replaced with culture supplemented with 2% FBS. Cell supernatants were harvested at the indicated time points post-infection. Infected cell lysates were washed with PBS and then used to extract RNA according to the protocol below.

### RNA extraction, reverse transcription and quantitative PCR

Viral RNA was extracted from the infected cells using RNA extraction kits (QIAGEN, RNeasy Mini Kit, cat. 74106) following the manufacturer’s instructions. Complementary DNA (cDNA) was synthesized in a reverse transcription step using different polarity specific primers. Primers for generating cDNA from segment 4 (HA) vRNA and mRNA were 5’-ACAGCCACAACGGAAAACTATG-3’ and Oligo-dT, respectively. Primers for generating cDNA from segment 7 (M) vRNA, cRNA, and mRNA were 5’-CTTGAAGATGTCTTTGCAGG-3’, 5’-AGCAGAAACAAGGTAGT-3’, and Oligo-dT, respectively. To quantify the vRNA, cRNA and mRNA levels, real-time quantitative PCR analysis with a gene specific primer pair using SYBR green PCR mix (Applied Biosystems) was performed and data was analyzed on the Applied Biosystems ViiATM 7 Real-Time PCR System. For HA vRNA and mRNA analysis, the following primers were used: forward primer, 5’-GGCCCAACCACAACACAAAC-3’, reverse primer, 5’-AGCCCTCCTTCTCCGTCAGC-3’. For M vRNA and mRNA analysis, the following primers were used: forward primer, 5’- CCAATCCTGTCACCTCTGAC-3’, reverse primer, 5’- TGGACAAAGCGTCTACGC-3’. β-actin was detected as a reference gene using the following primers: Forward primer, 5’-GTACGCCAACACAGTGCTG-3’, Reverse primer, 5’-CGTCATACTCCTGCTTGCTG-3’ [82]. The gene expression was calculated by normalizing target gene expression to β-actin for each sample and expressed as 2^ΔCt^. Analyses were performed using 7500 Fast System SDS software (Applied Biosystems).

### Chemokine and cytokine detection

Cytokine quantities for IL-6, TNF-a, IL-10, CXCL1, IL-12p40, and IFN-g in the lung tissue were determined by the mouse proinflammatory 7-plex tissue culture kit (Meso Scale Discovery, cat K15012B-1) using 25ul of homogenized lung tissue according to manufacturer’s instructions. The concentration of IFN-α and IFN-β from mouse cell supernatant and mouse lung tissue was measured with VeriKine mouse IFN-α ELISA kit (PBL, cat 42400) and VeriKine mouse IFN-β ELISA kit (PBL, cat 42400), respectively.

The mRNA level of TNF-α, IFN-β, IL-6, and IFN-α in GM-DCs were tested with SYBR green method as described above. Primers used for the cytokine test were the following:

TNF-α-F, 5'-GGCAGGTCTACTTTGGAGTCATTG-3’, TNF-α-R, 5'-ACATTCGAGGCTCCAGTGAATTCGG-3’; IFN-β-F, 5'- AAGAGTTACACTGCCTTTGCCATC-3’, IFN-β-R, 5'- CACTGTCTGCTGGTGGAGTTCATC-3’; IL-6-F, 5'-GACAAAGCCAGAGTCCTTCAG AGAG-3’, IL-6-R, 5'-CTAGGTTTGCCGAGTAGATCTC-3’; IFN-α-F, 5'-CGCAGGAGAAGGTGGATGCCCAG-3’, IFN-α-R, 5'-CAGCACATTGGCAGAGGAAGACAGG-3’ [19].

### Immunohistochemistry

Six to eight week old BALB/c mice were infected with 10^4^ or 10^5^ PFU of either 6:2 Eng/09, or 6:2 Tky/05 virus intranasally. Two to three days post infection mice were culled; the lungs inflated with 1mL PBS, and placed in 4% PFA solution overnight. Lungs were embedded in paraffin, and mounted on slides by the Inflammation, Repair, and Development group, NHLI at Imperial College London.

For immunohistochemistry, The Francis Crick Experimental Histopathology STP used formalin fixed paraffin embedded sections that were de-waxed in xylene then dehydrated by passage through graded alcohols to water. For antigen retrieval, sections were microwaved in sodium citrate, pH 6 for 15 minutes and then transferred to PBS. Endogenous peroxidase was blocked using 1.6% hydrogen peroxide in PBS for 10 minutes followed by washing in distilled water.

Biotinylated goat anti-NP antibody was used as primary antibody (USA biological, cat I7650) diluted to 1:100 in 1% BSA and incubated for 1 hour at room temperature. Sections were washed in PBS prior to applying ABC (Vector Laboratories, cat PK-6100) for 30 minutes. Following washing in PBS, DAB solution was applied for 2–5 minutes with development of the colour reaction being monitored microscopically. Slides were washed in tap water, stained with a light haematoxylin, dehydrated, cleared and then mounted.

Images were obtained by an Olympus VS120 slide reader, and analyzed by Image J software.

### Immunofluorescence microscopy

GM-DCs were seeded on Poly-L-Lysine treated glass coverslips and incubated for overnight. The cells were infected with the indicated viruses at a MOI = 4. After 4h and 8h infection, the GM-DCs were fixed for 20 min with 4% paraformaldehyde, permeabilized for 5 min with 0.1% TritonX 100 (Sigma, cat X100RS-5G), and blocked with 5% BSA for 1h in room temperature (RT). Cells were incubated with NP-FITC antibody (ThermoFisher, cat D67J, diluted with 1% BSAS at 1:20) for 1h at RT. DNA was stained with 4’,6’-diamino-2phenylindole (DAPI) for 10 min. After washing with PBST, samples were mounted with Mowiol under a coverslip. Multiple images were obtained for each sample by a Zeiss Axiovert 40CFL, and analyzed by AxioVision SE64 Rel software. Nuclei and FITC positive cells were counted for each image.

### Fluorescent-Activated Cell Sorting (FACS)

Mice were sacrificed and the lungs perfused with PBS. To obtain lung leukocytes, lung lobes were collected into a C-Tube (Miltenyi Biotech) containing complete DMEM (cDMEM; supplemented with 10% fetal bovine serum, 2mM L-glutamine, 100U/ml penicillin and 100μg/ml streptomycin), Collagenase D (1mg/ml; Roche) and DNase I (30μg/ml; Invitrogen) and processed with a gentleMACS dissociator (Miltenyi Biotech) according to the manufacturer’s protocol. Shredded tissue was incubated for 1h at 37°C. After lysis of red blood cells, cells were strained through a 100μm filter (BD Bioscience). For CD45+ lung cell sorting cells were incubated for 20 min with a purified rat IgG2b anti-mouse CD16/CD32 receptor antibody (BD Bioscience) to block Fc binding. Cells were then stained with fluorochrome-conjugated antibodies against CD45 (30-F11, eFluor780, eBiosciences) in PBS containing 1% BSA and 5mM EDTA for 25 min at 4°C. 1ng/ml of Hoeschst 3358 (Pentahydrate (bis-Benzimide); Thermo Scientific) was added just before running the sample for exclusion of dead cells. Cells were sorted using a standard Becton Dickinson Aria-II and stored in RLT buffer until RNA extraction was performed. The purity of CD45+ cells was >98%.

### Statistical analysis

All data are presented as mean ± SD of three or more experiments. For viral replication kinetics and weight loss in Fig 2A and S10B Fig, area under the curve (AUC) for each virus was calculated. Difference in the AUC between viruses was analyzed with one-way ANOVA and Bonferroni’s multiple comparisons test. For weight loss of the NP mutant virus infected mice (Fig 7G), two-way ANOVA test with post-tests for multiple comparisons was performed to determine P-value. One-way ANOVA analysis was used for the other comparisons among groups. Pearson correlation test was performed for the correlation analysis. P-value<0.05 wasnconsidered significantly different. All data analyses and preparation of all graphs were carried out with GraphPad Prism (GraphPad Software, San Diego, CA).

### Safety/biosecurity

All work with infectious agents was conducted in biosafety level 2 facilities, approved by the Health and Safety Executive of the UK and in accordance with local rules, at Imperial College London, UK.

### Statement on animal ethics

All work was approved by the local genetic manipulation (GM) safety committee of Imperial College London, St. Mary’s Campus (centre number GM77), and the Health and Safety Executive of the United Kingdom and carried out in accordance with the approved guidelines. All animal research described in this study was approved and carried out under a United Kingdom Home Office License, PPL 70/7501 in accordance with the approved guidelines, under the Animals (Scientific Procedures) Act 1986 (ASPA).

## Supporting information

S1 Fig Area of lung infection is similar between 6:2 Tky/05 and 6:2 Eng/09 viruses. Infected lungs harvested 3 days after infection with 10^4^ PFU of the viruses indicated were analysed for viral infection by an anti-influenza NP antibody. Biotinylated goat-anti-NP antibody was used as a primary antibody, and developed by ABC, followed by haematoxylin counterstain. Areas stained brown indicate influenza NP positive regions in the panels. (A, B) Mock infected mouse lungs. (B, C) 6:2 Eng/09 virus infected lungs. (D, E) 6:2 Tky/05 virus infected lungs.
(TIF)

S2 Fig Weight loss of the mice is associated with cytokine production in lung tissue. AUC for the weight in each group of influenza virus infected mice (Fig 2A) was calculated. The association between the AUCs and cytokine level on Day 2 were analyzed with Pearson correlation test. Red refers the AUC of weight curve of 6:2 Tky/05 virus infected mice; Yellow for 6:2 Vic/75 virus infected mice; Blue for 6:2 Eng/09 virus infected mice Green for 5:1:2 Eng09/TkyNS virus infected mice.
(TIF)

S3 Fig Dynamic cytokine and chemokine expression profile in the lung tissue early after infection. Six to eight week old female BALB/c mice (n = 20 per group) were infected i.n. with 10^4^ PFU RG viruses. At each indicated time point in each group, lungs of five mice were harvested and homogenized. Cytokine and chemokine protein level was determined by MSD or ELISA analysis. Bars represent mean ± SD (n = 5). Blue *, 6:2 Tky/05 vs. 6:2 Eng/09; orange *, Tky/05 vs. 6:2 Vic/75. *P<0.05, ** P<0.01, *** P<0.001, **** P<0.001; dpi, days post infection. Most of the cytokines tested at 1dpi were below the minimum level of detection (the dashed line in the figures), so statistic al analysis was not performed.
(TIF)

S4 Fig IFN-α, IFN-β and other cytokines production from bone marrow derived GM-DCs tested by qRT-PCR. Cytokine induction in GM-DCs (bone marrow derived dendritic cells propagated using GM-CSF) at 8 hpi (MOI = 10). Bars represent mean ± SD (n = 3). *P<0.05, **P<0.01, ***P<0.001, **** P<0.0001 indicate significant difference between 6:2 Tky/05 vs. 6:2 Eng/09, 6:2 Tky/05 vs. 6:2 Vic/75, as well as 6:2 Eng/09 vs. Eng/09:Tky/NS infected cells. hpi, hours post infection.
(TIFF)

S5 Fig IFN-β and IFN-α induction in FL-DCs (BMDC propagated using Flt3 ligand) after infection with reassortant influenza viruses. IFN-α/β induction in FL-DCs at 24hpi (MOI = 10). Bars show mean ± SD. The value of 6:2 Tky/05 group was compared with that of 6:2 Eng/09 or 6:2 Vic/75 groups. Statistical significance of difference between 6:2 Eng/09 and Eng/09:Tky/NS groups was also assessed.***P<0.001, **** P<0.0001. The dashed line indicates the lower detection limit.
(TIF)

S6 Fig Viral replication in A549 and LA4 cells. (A, B) A549 and (C, D) LA4 cells were infected with RG virus at MOI = 10. m and vRNA level in these cells at 2, 4 and 8 hpi were quantified with SYBR Green. Values were calculated by the 2^ΔCt^ method with β-actin as the control. Bars represent mean ± SD (n = 3). Blue *, 6:2 Tky/05 vs. 6:2 Eng/09; orange *, Tky/05 vs. 6:2 Vic/75; green *, 6:2 Eng/09 vs. Eng/09:Tky/NS. ** P<0.01, ****<0.0001. hpi, hours post infection.
(TIFF)

S7 Fig Surface protein from PR8 did not enhance virus’ ability to enter GM-DCs. GM-DCs were infected with the whole PR8 or PR8:TkyHA_sb_NA virus at MOI = 4. Cells were fixed at 4hpi and 8hpi, respectively. Nuclei were stained with DAPI (blue) and virus infected cells were stained for nucleoprotein (NP; green). Representative images are shown and the percentage of NP-positive cells was calculated. Bars represent mean ± SD. *P<0.05.
(TIFF)

S8 Fig IFN-α production from GM-DCs requires MAVS. Bone marrow derived GM-DCs from wild type C57/B6 (A) and MAVS knockout mice (B-D) were infected with the indicated RG viruses at MOI = 10, or treated with PolyIC. (A, B) Supernatant was collected at 24 hpi and IFN-α measured by ELISA. (C, D) m and vRNA level at 24 hpi were quantified with SYBR Green and the values were calculated by the 2^ΔCt^ method with β-actin as the control. Bars represent mean ± SD (n = 3). *P<0.05, **P<0.01, **** P<0.0001 indicate significant difference of 6:2 Tky/05 vs. 6:2 Eng/09, 6:2 Tky/05 vs. 6:2 Vic/75, as well as 6:2 Eng/09 vs. Eng/09:Tky/NS. The dashed line (A, B) indicates the minimum detection limit.
(TIFF)

S9 Fig Viral RNA, IFN-α and IL-6 transcripts detected in CD45 cells in vivo by qRT-PCR. 6–8 week old Balb/c mice were infected with 3x10^4^ (Black) or 10^5^ (Red) PFU of 6:2 Tky/05 virus or Mock control. CD45 cells were isolated from infected lungs 2 days post infection by FACS sorting.Total RNA was extracted from pooled CD45 positive cells, and qRT-PCR analysis was carried out for vRNA (A) mRNA (B) IFN-α (C) and IL-6 (D) transcripts. Data are displayed as relative expression compared to Mock calculated by 2^Δct^ to cellular β-actin. There are three technical replicates for each group and error is plotted as SD. ****P < 0.0001.
(TIFF)

S10 Fig Individual polymerase and NP genes of Tky/05 do not account for the high cytokine induction *in vivo*. (A) A panel of RG viruses with PR8 HA and NA gene segments combined with Tky/05 NS, polymerase and NP genes but where each polymerase or NP segment were exchanged for the equivalent segments from Eng09 virus was generated. (B) 6–8 week old Balb/c mice were infected with 10^5^ PFU each virus and weight loss monitored daily Lungs harvested from mice at day 2 post infection were assayed for viral titre (C) and cytokines IFN-α, IL-6, and TNF-α (D). **P<0.01, ***P<0.01, **** P<0.0001.
(TIFF)

## References

[1] Wang C, Yu H, Horby PW, Cao B, Wu P, Yang S, et al Comparison of patients hospitalized with influenza A subtypes H7N9, H5N1, and 2009 pandemic H1N1. *Clin Infect Dis*. 2014; 58(8):1095–1103. doi: 10.1093/cid/ciu053 . 24488975 10.1093/cid/ciu053 PMC3967826

[2] Zhang YH, Zhao Y, Li N, Peng YC, Giannoulatou E, Jin RH, et al Interferon-induced transmembrane protein-3 genetic variant rs12252-C is associated with severe influenza in Chinese individuals. *Nat Commun*. 2013; 4(1418 doi: 10.1038/ncomms2433 . 23361009 10.1038/ncomms2433 PMC3562464

[3] Hagan M, Ranadheera C, Audet J, Morin J, Leung A, Kobasa D. Post-exposure treatment with whole inactivated H5N1 avian influenza virus protects against lethal homologous virus infection in mice. *Sci Rep*. 2016; 6(29433 doi: 10.1038/srep29433 . 27405487 10.1038/srep29433 PMC4942574

[4] Sridhar S, Begom S, Bermingham A, Hoschler K, Adamson W, Carman W, et al Cellular immune correlates of protection against symptomatic pandemic influenza. *Nat Med*. 2013; 19(10):1305–1312. doi: 10.1038/nm.3350 . 24056771 10.1038/nm.3350

[5] Watanabe T, Kawaoka Y. Pathogenesis of the 1918 pandemic influenza virus. *PLoS Pathog*. 2011; 7(1):e1001218 doi: 10.1371/journal.ppat.1001218 . 21298032 10.1371/journal.ppat.1001218 PMC3029258

[6] Cumulative number of confirmed human cases of avian influenza A(H5N1) reported to WHO. Retrieved 10 February, 2017

[7] de Jong MD, Simmons CP, Thanh TT, Hien VM, Smith GJ, Chau TN, et al Fatal outcome of human influenza A (H5N1) is associated with high viral load and hypercytokinemia. *Nat Med*. 2006; 12(10):1203–1207. doi: 10.1038/nm1477 . 16964257 10.1038/nm1477 PMC4333202

[8] Chan MC, Cheung CY, Chui WH, Tsao SW, Nicholls JM, Chan YO, et al Proinflammatory cytokine responses induced by influenza A (H5N1) viruses in primary human alveolar and bronchial epithelial cells. *Respir Res*. 2005; 6(135 doi: 10.1186/1465-9921-6-135 . 16283933 10.1186/1465-9921-6-135 PMC1318487

[9] Peiris JS, Yu WC, Leung CW, Cheung CY, Ng WF, Nicholls JM, et al Re-emergence of fatal human influenza A subtype H5N1 disease. *Lancet*. 2004; 363(9409):617–619. doi: 10.1016/S0140-6736(04)15595-5 . 14987888 10.1016/S0140-6736(04)15595-5 PMC7112424

[10] Cheung CY, Poon LL, Lau AS, Luk W, Lau YL, Shortridge KF, et al Induction of proinflammatory cytokines in human macrophages by influenza A (H5N1) viruses: a mechanism for the unusual severity of human disease? *Lancet*. 2002; 360(9348):1831–1837. . 12480361 10.1016/s0140-6736(02)11772-7

[11] Hui KP, Lee SM, Cheung CY, Ng IH, Poon LL, Guan Y, et al Induction of proinflammatory cytokines in primary human macrophages by influenza A virus (H5N1) is selectively regulated by IFN regulatory factor 3 and p38 MAPK. *J Immunol*. 2009; 182(2):1088–1098. . 19124752 10.4049/jimmunol.182.2.1088

[12] Abdel-Ghafar AN, Chotpitayasunondh T, Gao Z, Hayden FG, Nguyen DH, de Jong MD, et al Update on avian influenza A (H5N1) virus infection in humans. *N Engl J Med*. 2008; 358(3):261–273. doi: 10.1056/NEJMra0707279 . 18199865 10.1056/NEJMra0707279

[13] Mori M, Rothman AL, Kurane I, Montoya JM, Nolte KB, Norman JE, et al High levels of cytokine-producing cells in the lung tissues of patients with fatal hantavirus pulmonary syndrome. *J Infect Dis*. 1999; 179(2):295–302. doi: 10.1086/314597 . 9878011 10.1086/314597

[14] Safronetz D, Prescott J, Feldmann F, Haddock E, Rosenke R, Okumura A, et al Pathophysiology of hantavirus pulmonary syndrome in rhesus macaques. *Proc Natl Acad Sci U S A*. 2014; 111(19):7114–7119. doi: 10.1073/pnas.1401998111 . 24778254 10.1073/pnas.1401998111 PMC4024883

[15] Rothman AL. Immunity to dengue virus: a tale of original antigenic sin and tropical cytokine storms. *Nat Rev Immunol*. 2011; 11(8):532–543. doi: 10.1038/nri3014 . 21760609 10.1038/nri3014

[16] Imai M, Herfst S, Sorrell EM, Schrauwen EJ, Linster M, De Graaf M, et al Transmission of influenza A/H5N1 viruses in mammals. *Virus Res*. 2013; 178(1):15–20. doi: 10.1016/j.virusres.2013.07.017 . 23954580 10.1016/j.virusres.2013.07.017 PMC3838911

[17] Herfst S, Schrauwen EJ, Linster M, Chutinimitkul S, de Wit E, Munster VJ, et al Airborne transmission of influenza A/H5N1 virus between ferrets. *Science*. 2012; 336(6088):1534–1541. doi: 10.1126/science.1213362 . 22723413 10.1126/science.1213362 PMC4810786

[18] Goritzka M, Makris S, Kausar F, Durant LR, Pereira C, Kumagai Y, et al Alveolar macrophage-derived type I interferons orchestrate innate immunity to RSV through recruitment of antiviral monocytes. *J Exp Med*. 2015; 212(5):699–714. doi: 10.1084/jem.20140825 . 25897172 10.1084/jem.20140825 PMC4419339

[19] Goritzka M, Durant LR, Pereira C, Salek-Ardakani S, Openshaw PJ, Johansson C. Alpha/beta interferon receptor signaling amplifies early proinflammatory cytokine production in the lung during respiratory syncytial virus infection. *J Virol*. 2014; 88(11):6128–6136. doi: 10.1128/JVI.00333-14 . 24648449 10.1128/JVI.00333-14 PMC4093897

[20] Davidson S, Crotta S, McCabe TM, Wack A. Pathogenic potential of interferon alphabeta in acute influenza infection. *Nat Commun*. 2014; 5(3864 doi: 10.1038/ncomms4864 . 24844667 10.1038/ncomms4864 PMC4033792

[21] Pulendran B, Maddur MS. Innate immune sensing and response to influenza. *Curr Top Microbiol Immunol*. 2015; 386(23–71. doi: 10.1007/82_2014_405 . 25078919 10.1007/82_2014_405 PMC4346783

[22] Iwasaki A, Pillai PS. Innate immunity to influenza virus infection. *Nat Rev Immunol*. 2014; 14(5):315–328. doi: 10.1038/nri3665 . 24762827 10.1038/nri3665 PMC4104278

[23] Rajsbaum R, Albrecht RA, Wang MK, Maharaj NP, Versteeg GA, Nistal-Villan E, et al Species-specific inhibition of RIG-I ubiquitination and IFN induction by the influenza A virus NS1 protein. *PLoS Pathog*. 2012; 8(11):e1003059 doi: 10.1371/journal.ppat.1003059 . 23209422 10.1371/journal.ppat.1003059 PMC3510253

[24] Loo YM, Fornek J, Crochet N, Bajwa G, Perwitasari O, Martinez-Sobrido L, et al Distinct RIG-I and MDA5 signaling by RNA viruses in innate immunity. *J Virol*. 2008; 82(1):335–345. doi: 10.1128/JVI.01080-07 . 17942531 10.1128/JVI.01080-07 PMC2224404

[25] Nemeroff ME, Barabino SM, Li Y, Keller W, Krug RM. Influenza virus NS1 protein interacts with the cellular 30 kDa subunit of CPSF and inhibits 3'end formation of cellular pre-mRNAs. *Mol Cell*. 1998; 1(7):991–1000. . 9651582 10.1016/s1097-2765(00)80099-4

[26] Hatada E, Fukuda R. Binding of influenza A virus NS1 protein to dsRNA in vitro. *J Gen Virol*. 1992; 73 (Pt 12)(3325–3329. doi: 10.1099/0022-1317-73-12-3325 1469370 10.1099/0022-1317-73-12-3325

[27] Wang W, Riedel K, Lynch P, Chien CY, Montelione GT, Krug RM. RNA binding by the novel helical domain of the influenza virus NS1 protein requires its dimer structure and a small number of specific basic amino acids. *Rna*. 1999; 5(2):195–205. . 10024172 10.1017/s1355838299981621 PMC1369752

[28] Jia D, Rahbar R, Chan RW, Lee SM, Chan MC, Wang BX, et al Influenza virus non-structural protein 1 (NS1) disrupts interferon signaling. *PLoS One*. 2010; 5(11):e13927 doi: 10.1371/journal.pone.0013927 . 21085662 10.1371/journal.pone.0013927 PMC2978095

[29] Menachery VD, Eisfeld AJ, Schafer A, Josset L, Sims AC, Proll S, et al Pathogenic influenza viruses and coronaviruses utilize similar and contrasting approaches to control interferon-stimulated gene responses. *MBio*. 2014; 5(3):e01174–01114. doi: 10.1128/mBio.01174-14 . 24846384 10.1128/mBio.01174-14 PMC4030454

[30] Ludwig S, Schultz U, Mandler J, Fitch WM, Scholtissek C. Phylogenetic relationship of the nonstructural (NS) genes of influenza A viruses. *Virology*. 1991; 183(2):566–577. . 1830182 10.1016/0042-6822(91)90985-k

[31] Hayman A, Comely S, Lackenby A, Hartgroves LC, Goodbourn S, McCauley JW, et al NS1 proteins of avian influenza A viruses can act as antagonists of the human alpha/beta interferon response. *J Virol*. 2007; 81(5):2318–2327. doi: 10.1128/JVI.01856-06 P . 17182679 10.1128/JVI.01856-06 PMC1865923

[32] Wu NC, Young AP, Al-Mawsawi LQ, Olson CA, Feng J, Qi H, et al High-throughput identification of loss-of-function mutations for anti-interferon activity in the influenza A virus NS segment. *J Virol*. 2014; 88(17):10157–10164. doi: 10.1128/JVI.01494-14 . 24965464 10.1128/JVI.01494-14 PMC4136320

[33] Matthaei M, Budt M, Wolff T. Highly pathogenic H5N1 influenza A virus strains provoke heterogeneous IFN-alpha/beta responses that distinctively affect viral propagation in human cells. *PLoS One*. 2013; 8(2):e56659 doi: 10.1371/journal.pone.0056659 . 23451066 10.1371/journal.pone.0056659 PMC3581526

[34] Twu KY, Kuo RL, Marklund J, Krug RM. The H5N1 influenza virus NS genes selected after 1998 enhance virus replication in mammalian cells. *J Virol*. 2007; 81(15):8112–8121. doi: 10.1128/JVI.00006-07 . 17522219 10.1128/JVI.00006-07 PMC1951328

[35] Kawaoka Y, Webster RG. Sequence requirements for cleavage activation of influenza virus hemagglutinin expressed in mammalian cells. *Proc Natl Acad Sci U S A*. 1988; 85(2):324–328. . 2829180 10.1073/pnas.85.2.324 PMC279540

[36] Hatta M, Gao P, Halfmann P, Kawaoka Y. Molecular basis for high virulence of Hong Kong H5N1 influenza A viruses. *Science*. 2001; 293(5536):1840–1842. doi: 10.1126/science.1062882 . 11546875 10.1126/science.1062882

[37] Tundup S, Kandasamy M, Perez JT, Mena N. Endothelial cell tropism is a determinant of H5N1 pathogenesis in mammalian species. 2017; 13(3):e1006270 doi: 10.1371/journal.ppat.1006270 . 28282445 10.1371/journal.ppat.1006270 PMC5362246

[38] Tumpey TM, Basler CF, Aguilar PV, Zeng H, Solorzano A, Swayne DE, et al Characterization of the reconstructed 1918 Spanish influenza pandemic virus. *Science*. 2005; 310(5745):77–80. doi: 10.1126/science.1119392 . 16210530 10.1126/science.1119392

[39] Ramos I, Bernal-Rubio D, Durham N, Belicha-Villanueva A, Lowen AC, Steel J, et al Effects of receptor binding specificity of avian influenza virus on the human innate immune response. *J Virol*. 2011; 85(9):4421–4431. doi: 10.1128/JVI.02356-10 PMID:Pmc3126224. . 21345953 10.1128/JVI.02356-10 PMC3126224

[40] Sun X, Zeng H, Kumar A, Belser JA, Maines TR, Tumpey TM. Constitutively Expressed IFITM3 Protein in Human Endothelial Cells Poses an Early Infection Block to Human Influenza Viruses. *J Virol*. 2016; 90(24):11157–11167. doi: 10.1128/JVI.01254-16 . 27707929 10.1128/JVI.01254-16 PMC5126373

[41] Imai Y, Kuba K, Neely GG, Yaghubian-Malhami R, Perkmann T, van Loo G, et al Identification of oxidative stress and Toll-like receptor 4 signaling as a key pathway of acute lung injury. *Cell*. 2008; 133(2):235–249. doi: 10.1016/j.cell.2008.02.043 . 18423196 10.1016/j.cell.2008.02.043 PMC7112336

[42] Cline TD, Karlsson EA, Freiden P, Seufzer BJ, Rehg JE, Webby RJ, et al Increased pathogenicity of a reassortant 2009 pandemic H1N1 influenza virus containing an H5N1 hemagglutinin. *J Virol*. 2011; 85(23):12262–12270. doi: 10.1128/JVI.05582-11 . 21917948 10.1128/JVI.05582-11 PMC3209346

[43] Kobasa D, Takada A, Shinya K, Hatta M, Halfmann P, Theriault S, et al Enhanced virulence of influenza A viruses with the haemagglutinin of the 1918 pandemic virus. *Nature*. 2004; 431(7009):703–707. doi: 10.1038/nature02951 15470432 10.1038/nature02951

[44] Carlson CM, Turpin EA, Moser LA, O'Brien KB, Cline TD, Jones JC, et al Transforming growth factor-beta: activation by neuraminidase and role in highly pathogenic H5N1 influenza pathogenesis. *PLoS Pathog*. 2010; 6(10):e1001136 doi: 10.1371/journal.ppat.1001136 P . 20949074 10.1371/journal.ppat.1001136 PMC2951376

[45] Goto H, Kawaoka Y. A novel mechanism for the acquisition of virulence by a human influenza A virus. *Proc Natl Acad Sci U S A*. 1998; 95(17):10224–10228. . 9707628 10.1073/pnas.95.17.10224 PMC21489

[46] Hessel A, Savidis-Dacho H, Coulibaly S, Portsmouth D, Kreil TR, Crowe BA, et al MVA vectors expressing conserved influenza proteins protect mice against lethal challenge with H5N1, H9N2 and H7N1 viruses. *PLoS One*. 2014; 9(2):e88340 doi: 10.1371/journal.pone.0088340 . 24523886 10.1371/journal.pone.0088340 PMC3921149

[47] Weinheimer VK, Becher A, Tonnies M, Holland G, Knepper J, Bauer TT, et al Influenza A viruses target type II pneumocytes in the human lung. *J Infect Dis*. 2012; 206(11):1685–1694. doi: 10.1093/infdis/jis455 . 22829640 10.1093/infdis/jis455 PMC7107318

[48] Knepper J, Schierhorn KL, Becher A, Budt M, Tonnies M, Bauer TT, et al The novel human influenza A(H7N9) virus is naturally adapted to efficient growth in human lung tissue. *MBio*. 2013; 4(5):e00601–00613. doi: 10.1128/mBio.00601-13 PMID:Pmc3791893. . 24105764 10.1128/mBio.00601-13 PMC3791893

[49] Matrosovich MN, Matrosovich TY, Gray T, Roberts NA, Klenk HD. Human and avian influenza viruses target different cell types in cultures of human airway epithelium. *Proc Natl Acad Sci U S A*. 2004; 101(13):4620–4624. doi: 10.1073/pnas.0308001101 . 15070767 10.1073/pnas.0308001101 PMC384796

[50] Shelton H, Smith M, Hartgroves L, Stilwell P, Roberts K, Johnson B, et al An influenza reassortant with polymerase of pH1N1 and NS gene of H3N2 influenza A virus is attenuated in vivo. *J Gen Virol*. 2012; 93(Pt 5):998–1006. doi: 10.1099/vir.0.039701-0 . 22323532 10.1099/vir.0.039701-0 PMC3541804

[51] Kallfass C, Lienenklaus S, Weiss S, Staeheli P. Visualizing the beta interferon response in mice during infection with influenza A viruses expressing or lacking nonstructural protein 1. *J Virol*. 2013; 87(12):6925–6930. doi: 10.1128/JVI.00283-13 . 23576514 10.1128/JVI.00283-13 PMC3676098

[52] Cella M, Jarrossay D, Facchetti F, Alebardi O, Nakajima H, Lanzavecchia A, et al Plasmacytoid monocytes migrate to inflamed lymph nodes and produce large amounts of type I interferon. *Nat Med*. 1999; 5(8):919–923. doi: 10.1038/11360 10426316 10.1038/11360

[53] Ciancanelli MJ, Huang SX, Luthra P, Garner H, Itan Y, Volpi S, et al Infectious disease. Life-threatening influenza and impaired interferon amplification in human IRF7 deficiency. *Science*. 2015; 348(6233):448–453. doi: 10.1126/science.aaa1578 . 25814066 10.1126/science.aaa1578 PMC4431581

[54] Alsharifi M, Mullbacher A, Regner M. Interferon type I responses in primary and secondary infections. *Immunol Cell Biol*. 2008; 86(3):239–245. doi: 10.1038/sj.icb.7100159 . 18180794 10.1038/sj.icb.7100159

[55] Helft J, Manicassamy B, Guermonprez P, Hashimoto D, Silvin A, Agudo J, et al Cross-presenting CD103+ dendritic cells are protected from influenza virus infection. *J Clin Invest*. 2012; 122(11):4037–4047. doi: 10.1172/JCI60659 . 23041628 10.1172/JCI60659 PMC3484433

[56] Langlois RA, Varble A, Chua MA, Garcia-Sastre A, tenOever BR. Hematopoietic-specific targeting of influenza A virus reveals replication requirements for induction of antiviral immune responses. *Proc Natl Acad Sci U S A*. 2012; 109(30):12117–12122. doi: 10.1073/pnas.1206039109 . 22778433 10.1073/pnas.1206039109 PMC3409765

[57] Thor SW, Nguyen H, Balish A, Hoang AN, Gustin KM, Nhung PT, et al Detection and Characterization of Clade 1 Reassortant H5N1 Viruses Isolated from Human Cases in Vietnam during 2013. *PLoS One*. 2015; 10(8):e0133867 doi: 10.1371/journal.pone.0133867 . 26244768 10.1371/journal.pone.0133867 PMC4526568

[58] Manabe T, Yamaoka K, Tango T, Binh NG, Co DX, Tuan ND, et al Chronological, geographical, and seasonal trends of human cases of avian influenza A (H5N1) in Vietnam, 2003–2014: a spatial analysis. *BMC Infect Dis*. 2016; 16(64 doi: 10.1186/s12879-016-1391-8 . 26847341 10.1186/s12879-016-1391-8 PMC4743110

[59] Mathur MB, Patel RB, Gould M, Uyeki TM, Bhattacharya J, Xiao Y, et al Seasonal patterns in human A (H5N1) virus infection: analysis of global cases. *PLoS One*. 2014; 9(9):e106171 doi: 10.1371/journal.pone.0106171 PMID:Pmc4162536. 25215608 10.1371/journal.pone.0106171 PMC4162536

[60] Bradley KC, Galloway SE, Lasanajak Y, Song X, Heimburg-Molinaro J, Yu H, et al Analysis of influenza virus hemagglutinin receptor binding mutants with limited receptor recognition properties and conditional replication characteristics. *J Virol*. 2011; 85(23):12387–12398. doi: 10.1128/JVI.05570-11 . 21917953 10.1128/JVI.05570-11 PMC3209400

[61] Kumari K, Gulati S, Smith DF, Gulati U, Cummings RD, Air GM. Receptor binding specificity of recent human H3N2 influenza viruses. *Virol J*. 2007; 4(42 doi: 10.1186/1743-422X-4-42 . 17490484 10.1186/1743-422X-4-42 PMC1876801

[62] Stevens J, Blixt O, Tumpey TM, Taubenberger JK, Paulson JC, Wilson IA. Structure and receptor specificity of the hemagglutinin from an H5N1 influenza virus. *Science*. 2006; 312(5772):404–410. doi: 10.1126/science.1124513 . 16543414 10.1126/science.1124513

[63] DuBois RM, Zaraket H, Reddivari M, Heath RJ, White SW, Russell CJ. Acid stability of the hemagglutinin protein regulates H5N1 influenza virus pathogenicity. *PLoS Pathog*. 2011; 7(12):e1002398 doi: 10.1371/journal.ppat.1002398 . 22144894 10.1371/journal.ppat.1002398 PMC3228800

[64] Jia N, Barclay WS, Roberts K, Yen HL, Chan RW, Lam AK, et al Glycomic characterization of respiratory tract tissues of ferrets: implications for its use in influenza virus infection studies. *J Biol Chem*. 2014; 289(41):28489–28504. doi: 10.1074/jbc.M114.588541 . 25135641 10.1074/jbc.M114.588541 PMC4192499

[65] Sakabe S, Takano R, Nagamura-Inoue T, Yamashita N, Nidom CA, Quynh Le M, et al Differences in cytokine production in human macrophages and in virulence in mice are attributable to the acidic polymerase protein of highly pathogenic influenza A virus subtype H5N1. *J Infect Dis*. 2013; 207(2):262–271. doi: 10.1093/infdis/jis523 PMID:Pmc3611767. 23042757 10.1093/infdis/jis523 PMC3611767

[66] Mok KP, Wong CH, Cheung CY, Chan MC, Lee SM, Nicholls JM, et al Viral genetic determinants of H5N1 influenza viruses that contribute to cytokine dysregulation. *J Infect Dis*. 2009; 200(7):1104–1112. doi: 10.1086/605606 . 19694514 10.1086/605606 PMC4028720

[67] Waddell SJ, Popper SJ, Rubins KH, Griffiths MJ, Brown PO, Levin M, et al Dissecting interferon-induced transcriptional programs in human peripheral blood cells. *PLoS One*. 2010; 5(3):e9753 doi: 10.1371/journal.pone.0009753 . 20339534 10.1371/journal.pone.0009753 PMC2842296

[68] Helft J, Bottcher JP, Chakravarty P, Zelenay S, Huotari J, Schraml BU, et al Alive but Confused: Heterogeneity of CD11c(+) MHC Class II(+) Cells in GM-CSF Mouse Bone Marrow Cultures. *Immunity*. 2016; 44(1):3–4. doi: 10.1016/j.immuni.2015.12.014 26789913 10.1016/j.immuni.2015.12.014

[69] Zeng H, Goldsmith C, Thawatsupha P, Chittaganpitch M, Waicharoen S, Zaki S, et al Highly pathogenic avian influenza H5N1 viruses elicit an attenuated type i interferon response in polarized human bronchial epithelial cells. *J Virol*. 2007; 81(22):12439–12449. doi: 10.1128/JVI.01134-07 . 17855549 10.1128/JVI.01134-07 PMC2169033

[70] Bao Y, Bolotov P, Dernovoy D, Kiryutin B, Zaslavsky L, Tatusova T, et al The influenza virus resource at the National Center for Biotechnology Information. *J Virol*. 2008; 82(2):596–601. doi: 10.1128/JVI.02005-07 PMID:Pmc2224563. 17942553 10.1128/JVI.02005-07 PMC2224563

[71] Rehwinkel J, Tan CP, Goubau D, Schulz O, Pichlmair A, Bier K, et al RIG-I detects viral genomic RNA during negative-strand RNA virus infection. *Cell*. 2010; 140(3):397–408. doi: 10.1016/j.cell.2010.01.020 . 20144762 10.1016/j.cell.2010.01.020

[72] Baum A, Sachidanandam R, Garcia-Sastre A. Preference of RIG-I for short viral RNA molecules in infected cells revealed by next-generation sequencing. *Proc Natl Acad Sci U S A*. 2010; 107(37):16303–16308. doi: 10.1073/pnas.1005077107 . 20805493 10.1073/pnas.1005077107 PMC2941304

[73] Killip MJ, Smith M, Jackson D, Randall RE. Activation of the interferon induction cascade by influenza a viruses requires viral RNA synthesis and nuclear export. *J Virol*. 2014; 88(8):3942–3952. doi: 10.1128/JVI.03109-13 . 24478437 10.1128/JVI.03109-13 PMC3993719

[74] Hayman A, Comely S, Lackenby A, Murphy S, McCauley J, Goodbourn S, et al Variation in the ability of human influenza A viruses to induce and inhibit the IFN-beta pathway. *Virology*. 2006; 347(1):52–64. doi: 10.1016/j.virol.2005.11.024 . 16378631 10.1016/j.virol.2005.11.024

[75] Long JS, Giotis ES, Moncorge O, Frise R, Mistry B, James J, et al Species difference in ANP32A underlies influenza A virus polymerase host restriction. *Nature*. 2016; 529(7584):101–104. doi: 10.1038/nature16474 . 26738596 10.1038/nature16474 PMC4710677

[76] Elleman CJ, Barclay WS. The M1 matrix protein controls the filamentous phenotype of influenza A virus. *Virology*. 2004; 321(1):144–153. doi: 10.1016/j.virol.2003.12.009 15033573 10.1016/j.virol.2003.12.009

[77] Langlois RA, Albrecht RA, Kimble B, Sutton T, Shapiro JS, Finch C, et al MicroRNA-based strategy to mitigate the risk of gain-of-function influenza studies. *Nat Biotechnol*. 2013; 31(9):844–847. doi: 10.1038/nbt.2666 . 23934176 10.1038/nbt.2666 PMC3808852

[78] Kumagai Y, Takeuchi O, Kato H, Kumar H, Matsui K, Morii E, et al Alveolar macrophages are the primary interferon-alpha producer in pulmonary infection with RNA viruses. *Immunity*. 2007; 27(2):240–252. doi: 10.1016/j.immuni.2007.07.013 17723216 10.1016/j.immuni.2007.07.013

[79] Gilliet M, Boonstra A, Paturel C, Antonenko S, Xu XL, Trinchieri G, et al The development of murine plasmacytoid dendritic cell precursors is differentially regulated by FLT3-ligand and granulocyte/macrophage colony-stimulating factor. *J Exp Med*. 2002; 195(7):953–958. doi: 10.1084/jem.20020045 . 11927638 10.1084/jem.20020045 PMC2193725

[80] Naik SH, Proietto AI, Wilson NS, Dakic A, Schnorrer P, Fuchsberger M, et al Cutting edge: generation of splenic CD8+ and CD8- dendritic cell equivalents in Fms-like tyrosine kinase 3 ligand bone marrow cultures. *J Immunol*. 2005; 174(11):6592–6597. 15905497 10.4049/jimmunol.174.11.6592

[81] Brawand P, Fitzpatrick DR, Greenfield BW, Brasel K, Maliszewski CR, De Smedt T. Murine plasmacytoid pre-dendritic cells generated from Flt3 ligand-supplemented bone marrow cultures are immature APCs. *J Immunol*. 2002; 169(12):6711–6719. 12471102 10.4049/jimmunol.169.12.6711

[82] Huang Q, Li L, Lin Z, Xu W, Han S, Zhao C, et al Identification of Preferentially Expressed Antigen of Melanoma as a Potential Tumor Suppressor in Lung Adenocarcinoma. *Med Sci Monit*. 2016; 22(1837–1842. doi: 10.12659/MSM.895642 . 27241212 10.12659/MSM.895642 PMC4913835

