# Supplementary figures and images for: Internal genes of a highly pathogenic H5N1 influenza virus determine high viral replication in myeloid cells and severe outcome of infection in mice
Source: PLoS Pathog. 2018 Jan 4;14(1):e1006821. doi: 10.1371/journal.ppat.1006821 (PMC5771632; doi:10.1371/journal.ppat.1006821)

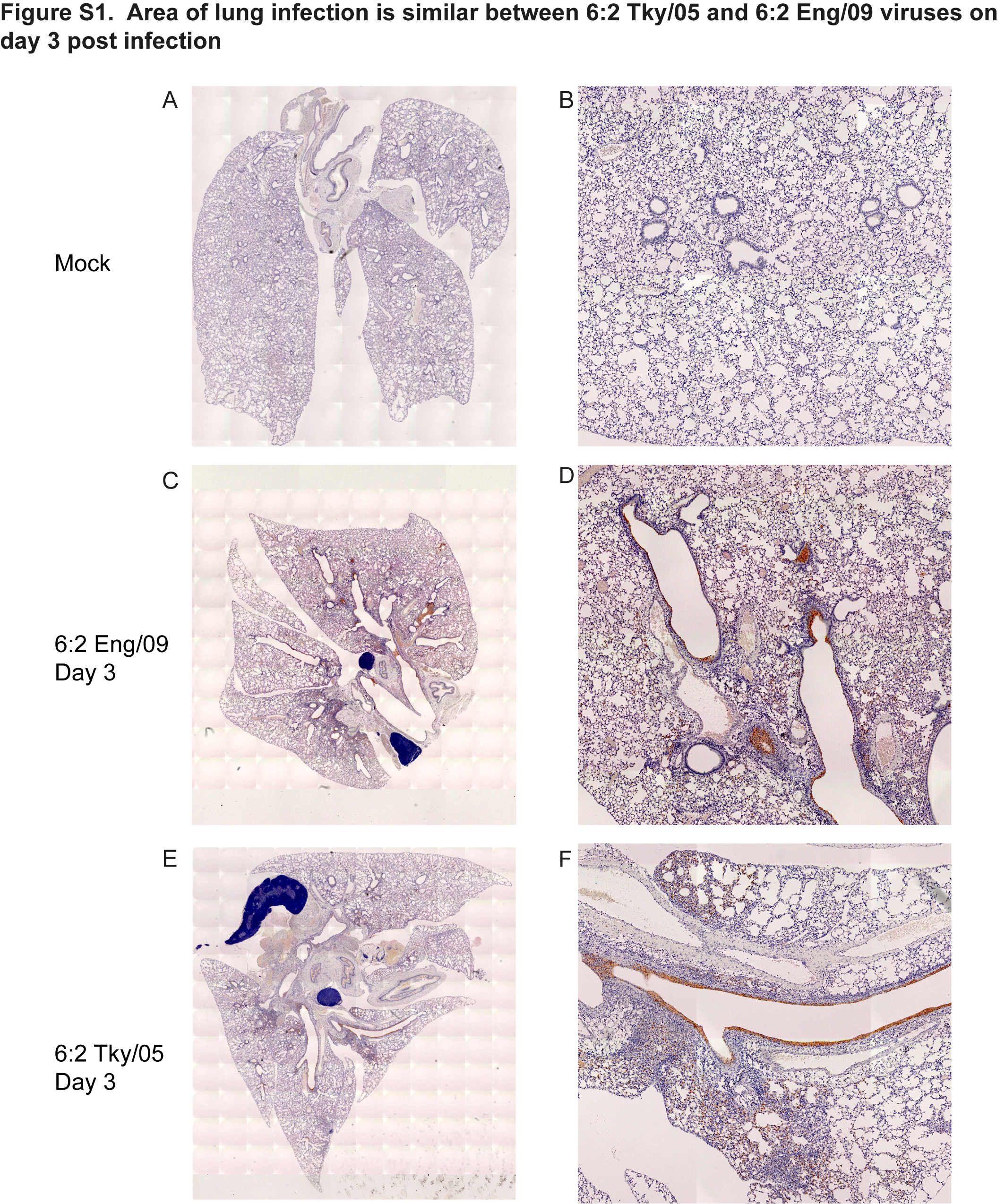

Supplement: S1 Fig — Infected lungs harvested 3 days after infection with 104 PFU of the viruses indicated were analysed for viral infection by an anti-influenza NP antibody. Biotinylated goat-anti-NP antibody was used as a primary antibody, and developed by ABC, followed by haematoxylin counterstain. Areas stained brown indicate influenza NP positive regions in the panels. (A, B) Mock infected mouse lungs. (B, C) 6:2 Eng/09 virus infected lungs. (D, E) 6:2 Tky/05 virus infected lungs. (TIF) [file ppat.1006821.s001.tif]

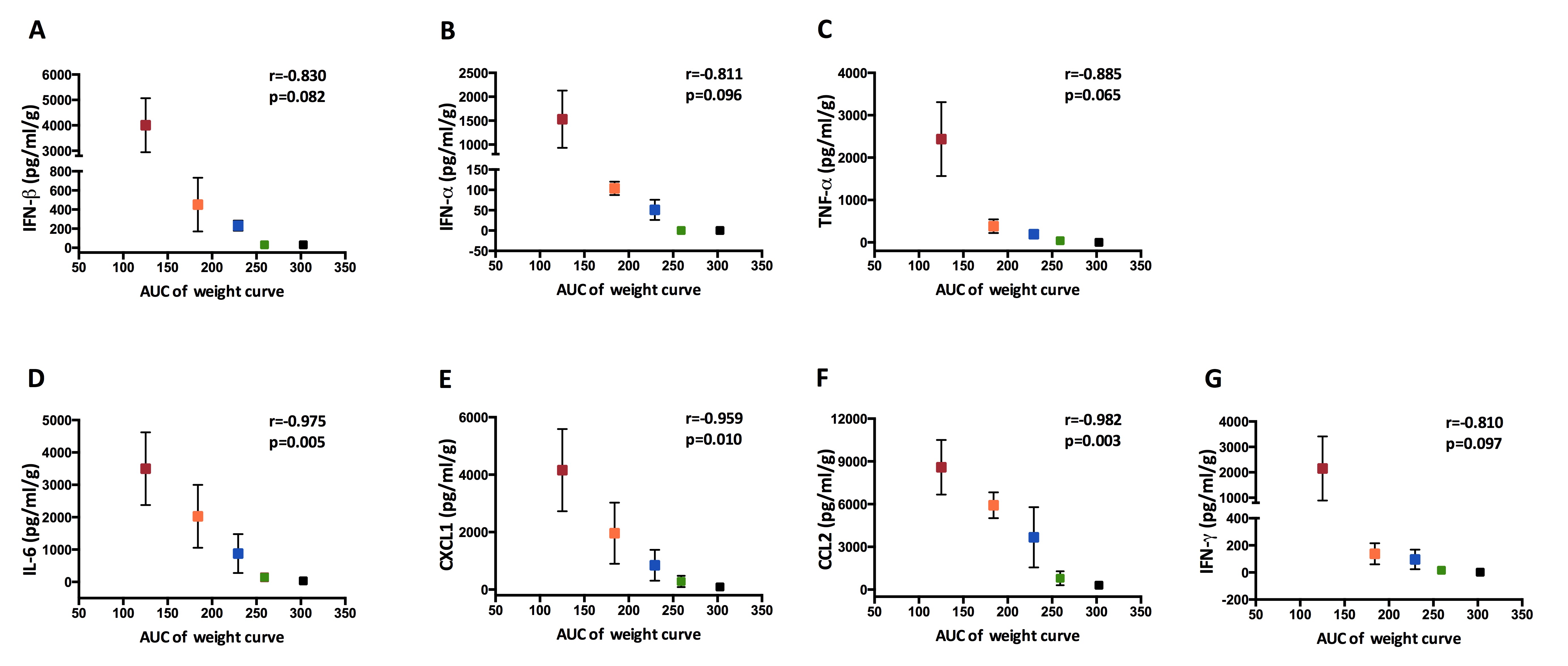

Supplement: S2 Fig — AUC for the weight in each group of influenza virus infected mice (Fig 2A) was calculated. The association between the AUCs and cytokine level on Day 2 were analyzed with Pearson correlation test. Red refers the AUC of weight curve of 6:2 Tky/05 virus infected mice; Yellow for 6:2 Vic/75 virus infected mice; Blue for 6:2 Eng/09 virus infected mice Green for 5:1:2 Eng09/TkyNS virus infected mice. (TIF) [file ppat.1006821.s002.tif]

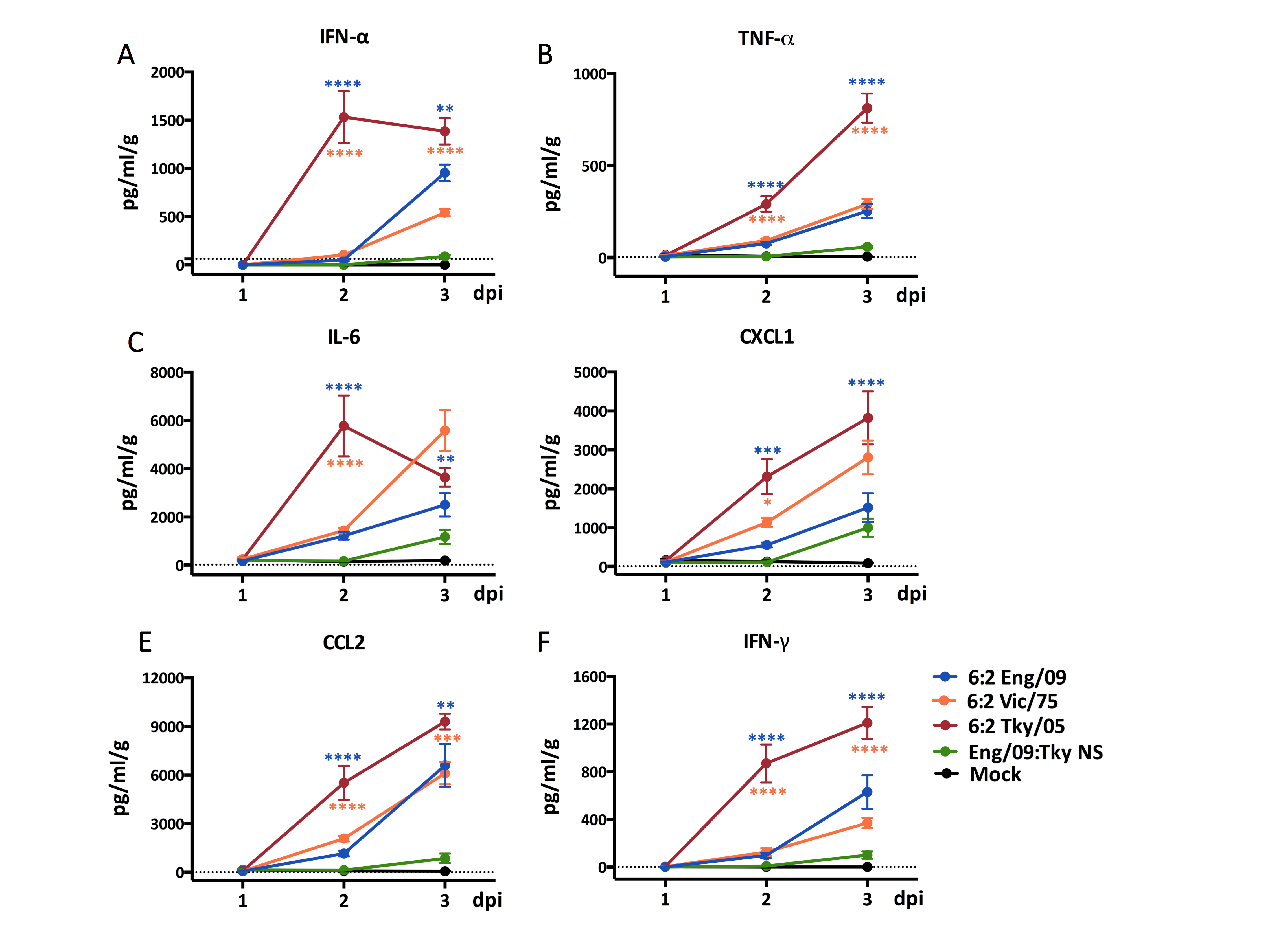

Supplement: S3 Fig — Six to eight week old female BALB/c mice (n = 20 per group) were infected i.n. with 104 PFU RG viruses. At each indicated time point in each group, lungs of five mice were harvested and homogenized. Cytokine and chemokine protein level was determined by MSD or ELISA analysis. Bars represent mean ± SD (n = 5). Blue *, 6:2 Tky/05 vs. 6:2 Eng/09; orange *, Tky/05 vs. 6:2 Vic/75. *P<0.05, ** P<0.01, *** P<0.001, **** P<0.001; dpi, days post infection. Most of the cytokines tested at 1dpi were below the minimum level of detection (the dashed line in the figures), so statistic al analysis was not performed. (TIF) [file ppat.1006821.s003.tif]

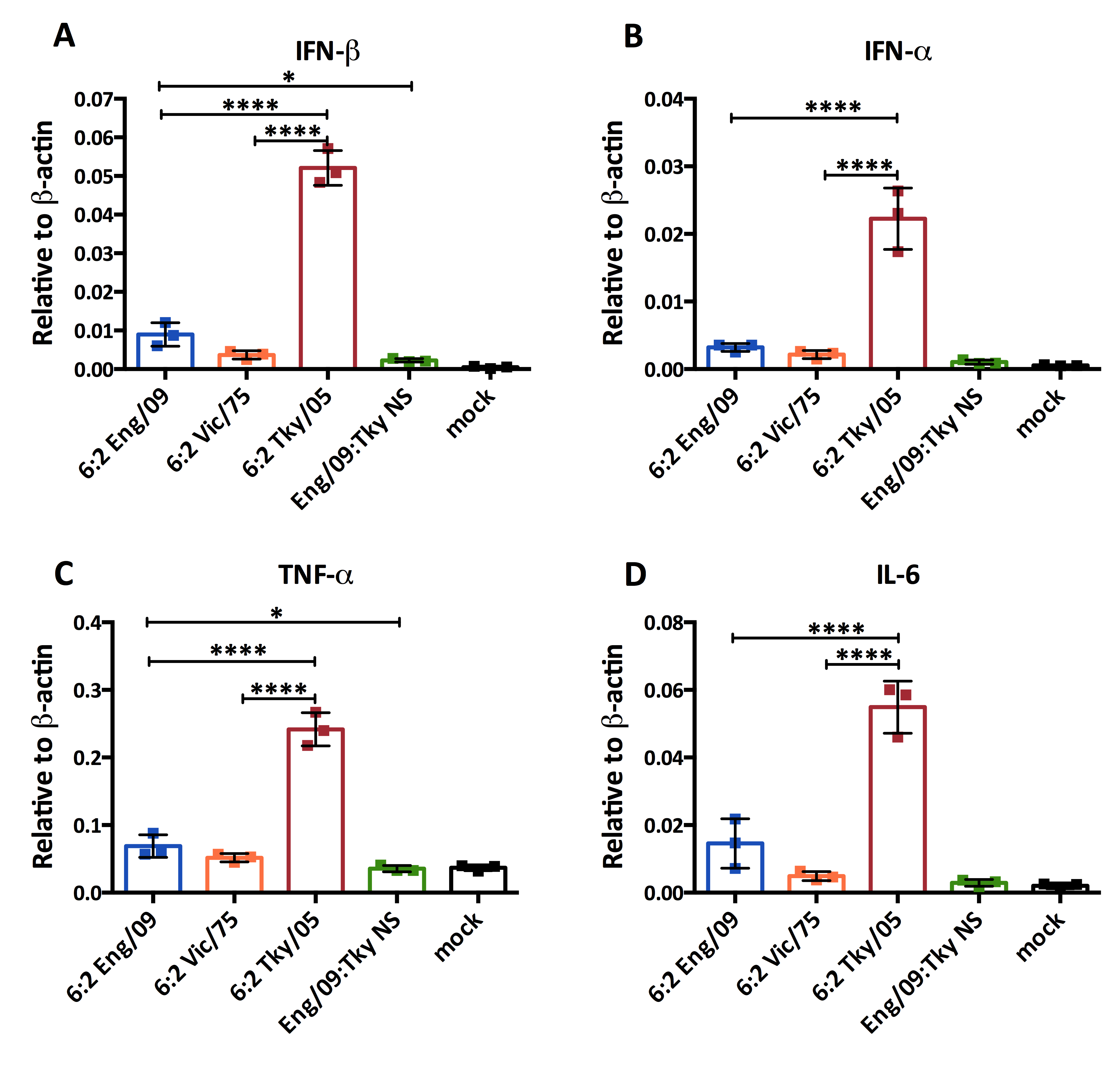

Supplement: S4 Fig — Cytokine induction in GM-DCs (bone marrow derived dendritic cells propagated using GM-CSF) at 8 hpi (MOI = 10). Bars represent mean ± SD (n = 3). *P<0.05, **P<0.01, ***P<0.001, **** P<0.0001 indicate significant difference between 6:2 Tky/05 vs. 6:2 Eng/09, 6:2 Tky/05 vs. 6:2 Vic/75, as well as 6:2 Eng/09 vs. Eng/09:Tky/NS infected cells. hpi, hours post infection. (TIFF) [file ppat.1006821.s004.tiff]

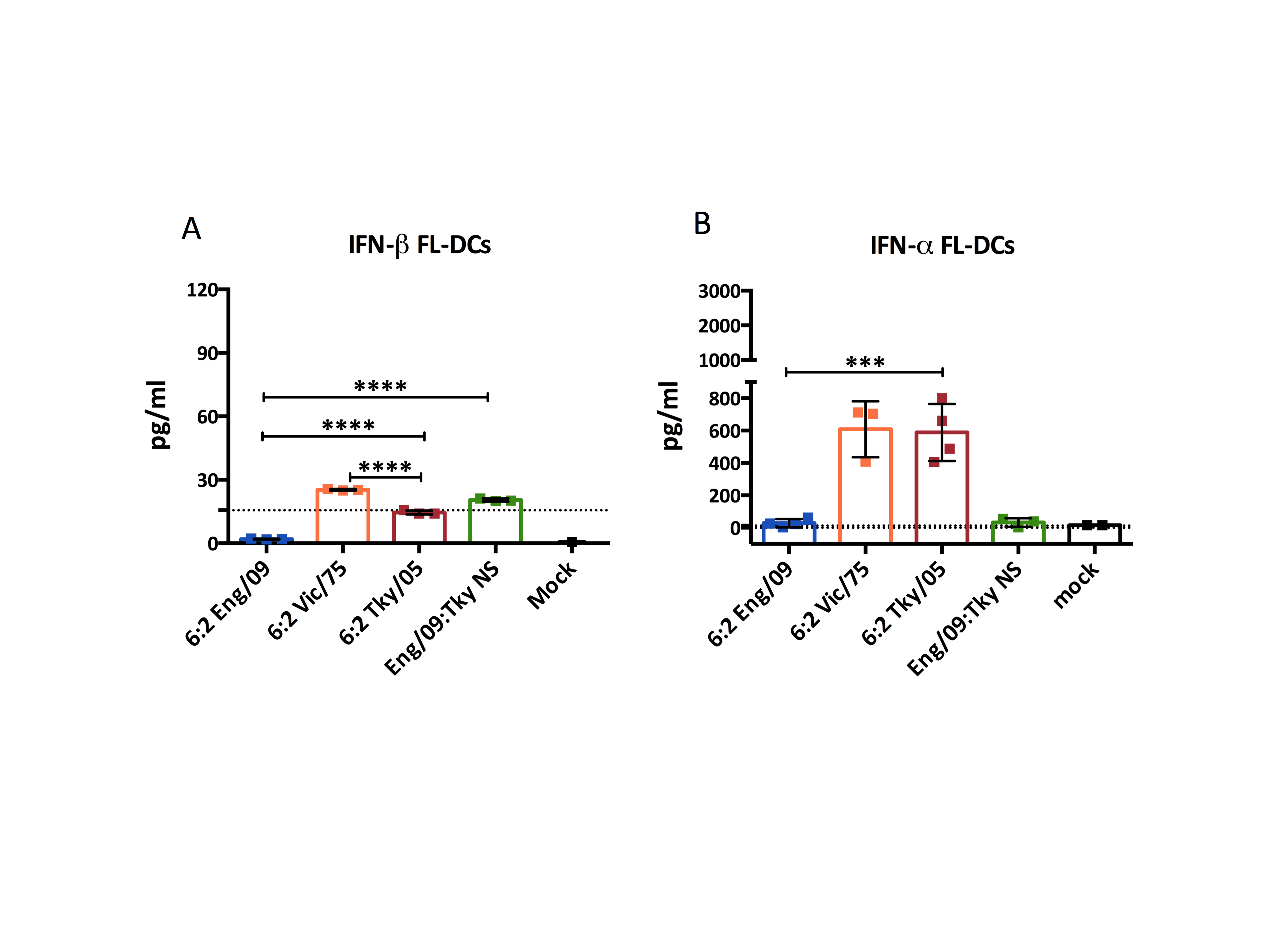

Supplement: S5 Fig — IFN-α/β induction in FL-DCs at 24hpi (MOI = 10). Bars show mean ± SD. The value of 6:2 Tky/05 group was compared with that of 6:2 Eng/09 or 6:2 Vic/75 groups. Statistical significance of difference between 6:2 Eng/09 and Eng/09:Tky/NS groups was also assessed.***P<0.001, **** P<0.0001. The dashed line indicates the lower detection limit. (TIF) [file ppat.1006821.s005.tif]

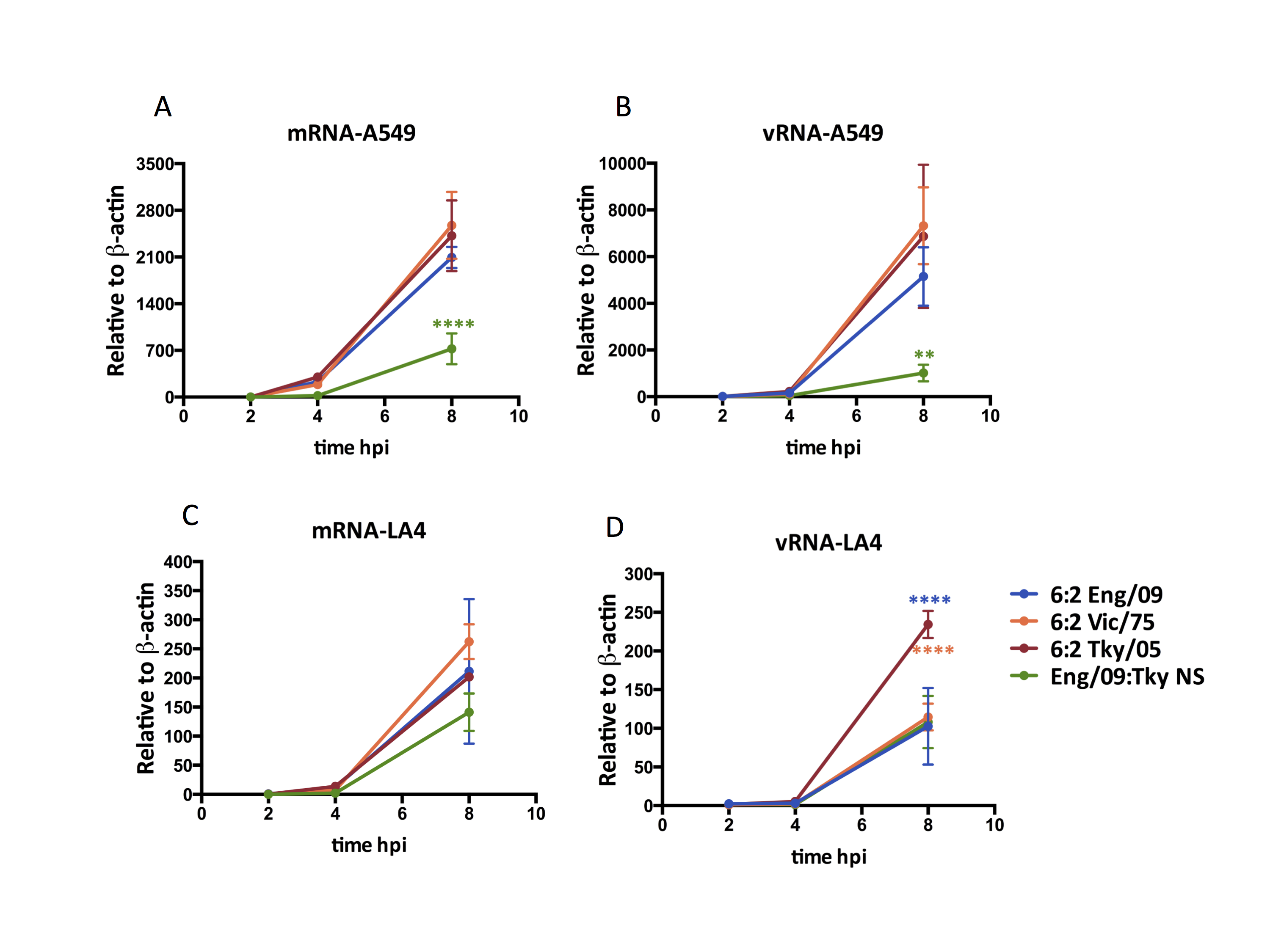

Supplement: S6 Fig — (A, B) A549 and (C, D) LA4 cells were infected with RG virus at MOI = 10. m and vRNA level in these cells at 2, 4 and 8 hpi were quantified with SYBR Green. Values were calculated by the 2ΔCt method with β-actin as the control. Bars represent mean ± SD (n = 3). Blue *, 6:2 Tky/05 vs. 6:2 Eng/09; orange *, Tky/05 vs. 6:2 Vic/75; green *, 6:2 Eng/09 vs. Eng/09:Tky/NS. ** P<0.01, ****<0.0001. hpi, hours post infection. (TIFF) [file ppat.1006821.s006.tiff]

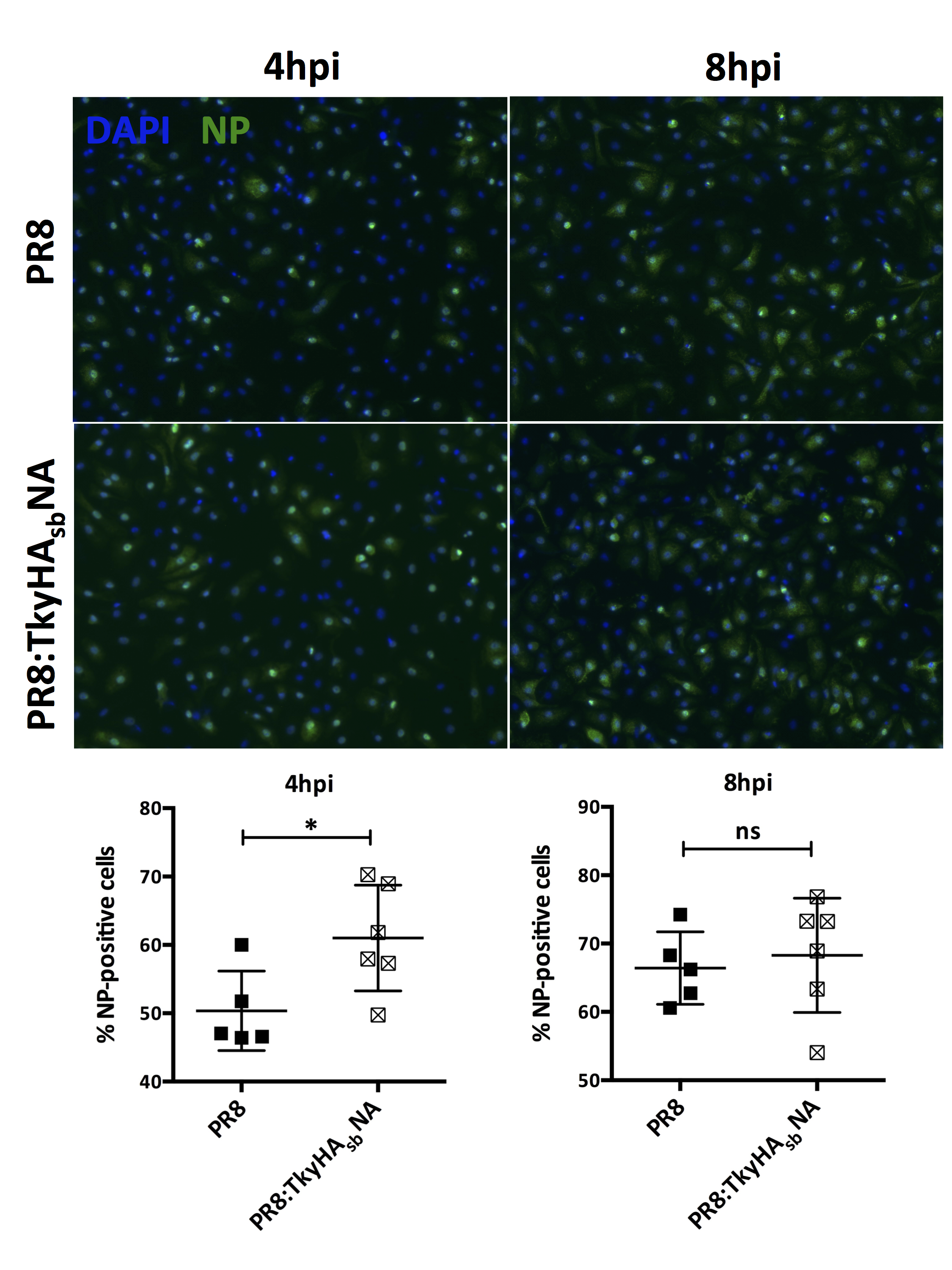

Supplement: S7 Fig — GM-DCs were infected with the whole PR8 or PR8:TkyHAsbNA virus at MOI = 4. Cells were fixed at 4hpi and 8hpi, respectively. Nuclei were stained with DAPI (blue) and virus infected cells were stained for nucleoprotein (NP; green). Representative images are shown and the percentage of NP-positive cells was calculated. Bars represent mean ± SD. *P<0.05. (TIFF) [file ppat.1006821.s007.tiff]

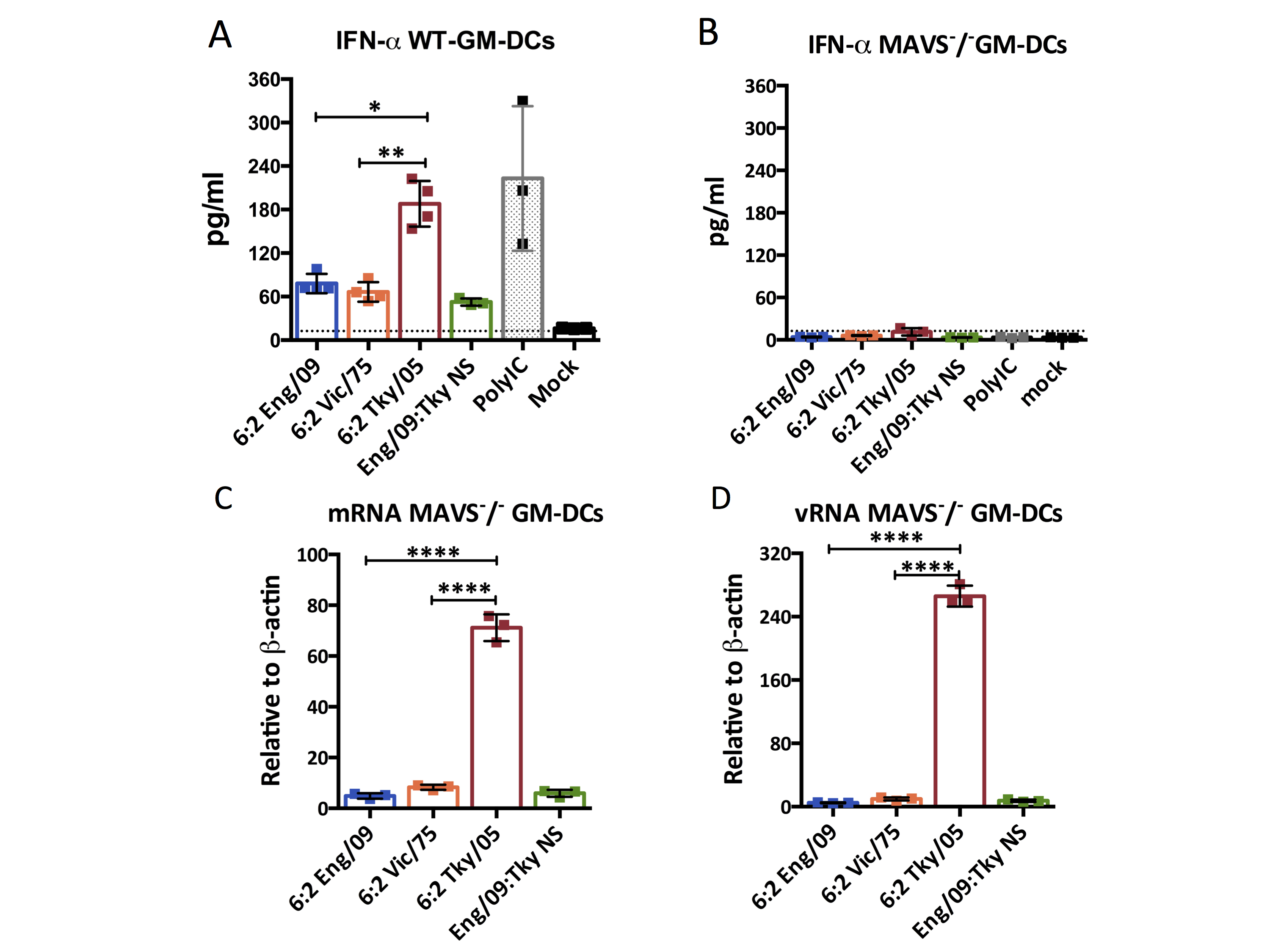

Supplement: S8 Fig — Bone marrow derived GM-DCs from wild type C57/B6 (A) and MAVS knockout mice (B-D) were infected with the indicated RG viruses at MOI = 10, or treated with PolyIC. (A, B) Supernatant was collected at 24 hpi and IFN-α measured by ELISA. (C, D) m and vRNA level at 24 hpi were quantified with SYBR Green and the values were calculated by the 2ΔCt method with β-actin as the control. Bars represent mean ± SD (n = 3). *P<0.05, **P<0.01, **** P<0.0001 indicate significant difference of 6:2 Tky/05 vs. 6:2 Eng/09, 6:2 Tky/05 vs. 6:2 Vic/75, as well as 6:2 Eng/09 vs. Eng/09:Tky/NS. The dashed line (A, B) indicates the minimum detection limit. (TIFF) [file ppat.1006821.s008.tiff]

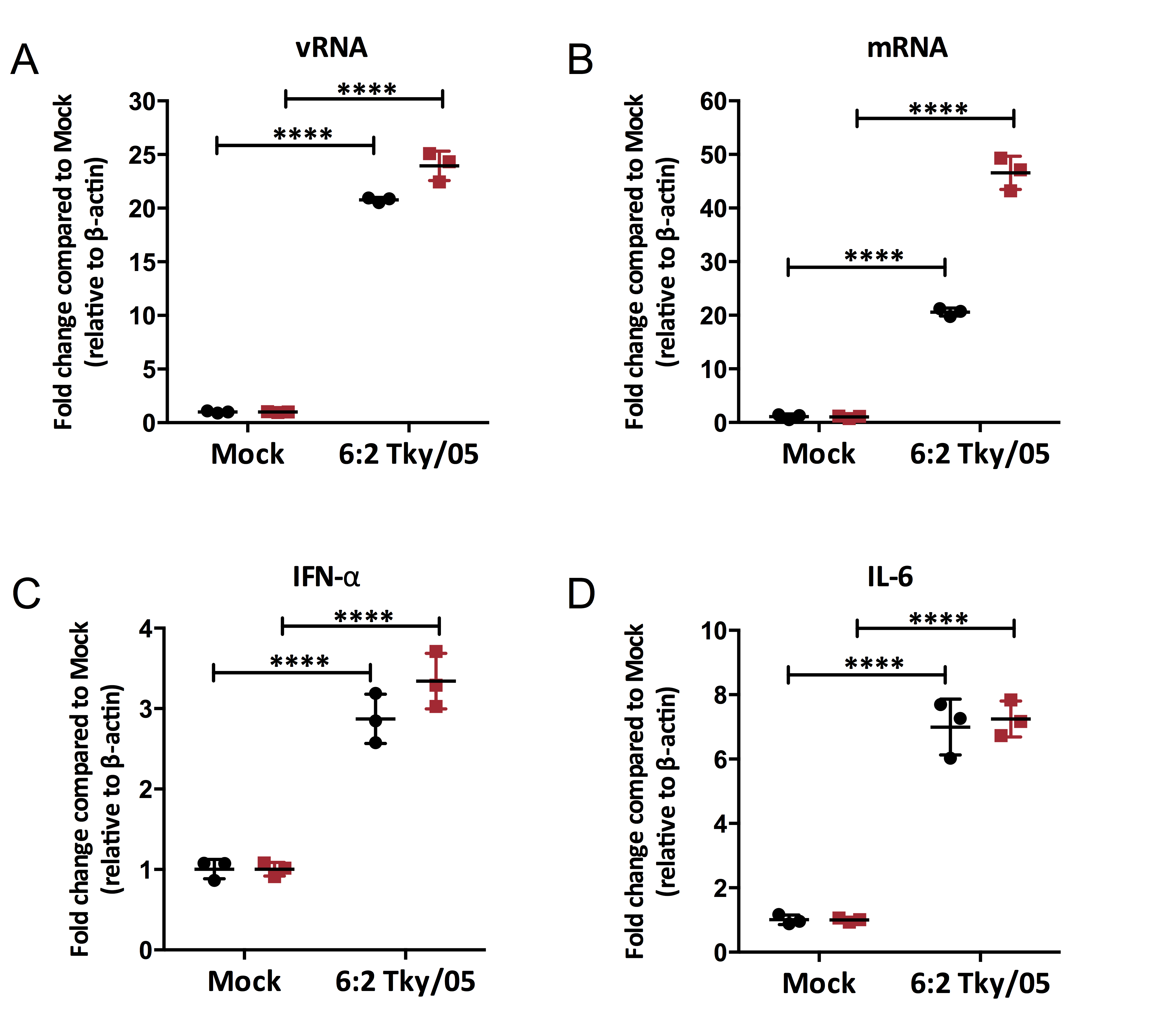

Supplement: S9 Fig — 6–8 week old Balb/c mice were infected with 3x10^4^ (Black) or 105 (Red) PFU of 6:2 Tky/05 virus or Mock control. CD45 cells were isolated from infected lungs 2 days post infection by FACS sorting.Total RNA was extracted from pooled CD45 positive cells, and qRT-PCR analysis was carried out for vRNA (A) mRNA (B) IFN-α (C) and IL-6 (D) transcripts. Data are displayed as relative expression compared to Mock calculated by 2Δct to cellular β-actin. There are three technical replicates for each group and error is plotted as SD. ****P < 0.0001. (TIFF) [file ppat.1006821.s009.tiff]

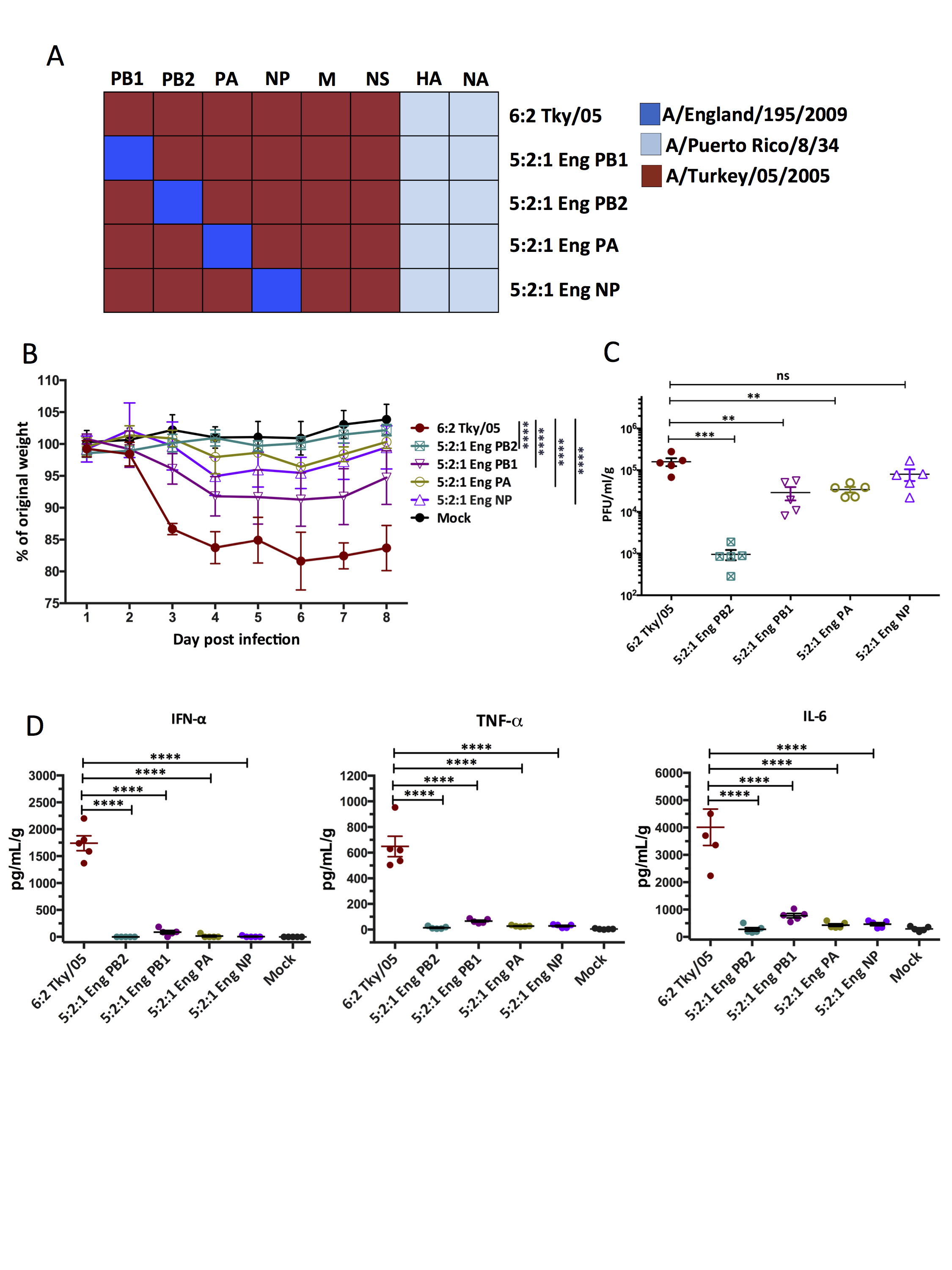

Supplement: S10 Fig — (A) A panel of RG viruses with PR8 HA and NA gene segments combined with Tky/05 NS, polymerase and NP genes but where each polymerase or NP segment were exchanged for the equivalent segments from Eng09 virus was generated. (B) 6–8 week old Balb/c mice were infected with 105 PFU each virus and weight loss monitored daily Lungs harvested from mice at day 2 post infection were assayed for viral titre (C) and cytokines IFN-α, IL-6, and TNF-α (D). **P<0.01, ***P<0.01, **** P<0.0001. (TIFF) [file ppat.1006821.s010.tiff]
